# Global Chromatin Domain Organization of the *Drosophila* Genome

**DOI:** 10.1371/journal.pgen.1000045

**Published:** 2008-03-28

**Authors:** Elzo de Wit, Ulrich Braunschweig, Frauke Greil, Harmen J. Bussemaker, Bas van Steensel

**Affiliations:** 1 Department of Molecular Biology, Netherlands Cancer Institute, Amsterdam, The Netherlands; 2 Department of Biological Sciences, Columbia University, New York, New York, United States of America; The Babraham Institute, United Kingdom

## Abstract

In eukaryotes, neighboring genes can be packaged together in specific chromatin structures that ensure their coordinated expression. Examples of such multi-gene chromatin domains are well-documented, but a global view of the chromatin organization of eukaryotic genomes is lacking. To systematically identify multi-gene chromatin domains, we constructed a compendium of genome-scale binding maps for a broad panel of chromatin-associated proteins in *Drosophila melanogaster*. Next, we computationally analyzed this compendium for evidence of multi-gene chromatin domains using a novel statistical segmentation algorithm. We find that at least 50% of all fly genes are organized into chromatin domains, which often consist of dozens of genes. The domains are characterized by various known and novel combinations of chromatin proteins. The genes in many of the domains are coregulated during development and tend to have similar biological functions. Furthermore, during evolution fewer chromosomal rearrangements occur inside chromatin domains than outside domains. Our results indicate that a substantial portion of the *Drosophila* genome is packaged into functionally coherent, multi-gene chromatin domains. This has broad mechanistic implications for gene regulation and genome evolution.

## Author Summary

Genes are packaged into chromatin by a variety of specialized proteins. Many different types of chromatin exist, and each may regulate gene expression in different ways. It was previously observed that neighboring genes are sometimes packaged together into a single type of chromatin, which can facilitate their coordinated regulation. However, it has been unclear whether such multi-gene chromatin domains are exceptional, or may occur more frequently. Here, we report a systematic analysis of genome-wide binding patterns of a large set of chromatin components in the fruit fly *Drosophila melanogaster*. Strikingly, we find that at least 50% of all genes in this organism are packaged together with several of their neighboring genes into a single type of chromatin. Each chromatin domain can include dozens of genes and can be made up of different combinations of chromatin proteins. We show that genes in each domain often have similar functions and are coordinately expressed during development. Moreover, we find that many of these multi-gene domains have been kept intact during evolution, indicating that they are important functional units. In summary, multi-gene chromatin domains are much more common than previously thought, and they are likely to play important roles in the orchestration of gene expression.

## Introduction

It is becoming increasingly clear that the ordering of genes in metazoan genomes is non-random [1],[2]. Functionally related genes are often located next to one another in the linear genome, and this proximity can be essential for their coordinated regulation during development [3]. Well-studied examples of this are the *β-globin* gene locus [4] and the *hox* gene clusters [5],[6]. Genome-scale studies point at the existence of many more clusters of functionally related genes [7]–[9]. In addition, analysis of transcriptome datasets has shown that genes with a similar expression pattern are frequently located in clusters in the genome. For example, testis- and sperm-specific genes in *Drosophila melanogaster* [10],[11] and muscle-specific genes in *Caenorhabditis elegans* [12] are significantly clustered. Analysis of genome-wide expression profiles during *Drosophila* development has identified many clusters of coexpressed neighboring genes, ranging from 10 to 30 genes in size [13]. Furthermore, the human genome shows large regions in which most genes are expressed at high levels, alternating with regions that contain predominantly lowly expressed genes [14],[15]. These observations strongly suggest that juxtaposition of genes in the linear genome can facilitate their coordinated regulation. However, the underlying molecular mechanisms are poorly understood.

Chromatin is a principal orchestrator of transcription. Neighboring genes can be packaged together into a single chromatin domain that may act as a regulatory unit [2],[3],[16],[17]. Several chromatin domains have been characterized in detail in a variety of species [18]–[23]. However, it remains unclear whether such domains are relatively rare, or represent a general principle of genome organization. Here, we present a systematic survey of chromatin domain organization of the *D. melanogaster* genome by computational analysis of a broad panel of genome-wide chromatin protein binding maps. Our results demonstrate that at least half of the Drosophila genome consists of multi-gene chromatin domains. Strikingly, these domains can be very large and include dozens of genes. We provide evidence that most of the newly identified domains are of functional relevance.

## Results

### A Compendium of Chromatin Protein Binding Maps

To systematically identify chromatin domains, we assembled a compendium of genome-scale binding maps of 29 broadly selected *Drosophila* chromatin components (Dataset S1). We included previously published DamID and ChIP-on-chip datasets [21], [22], [24]–[28] as well as newly generated DamID maps for 11 proteins (see Methods). The full list consists of heterochromatin proteins, Polycomb group proteins, chromatin remodeling proteins, high mobility group (HMG) proteins, various DNA binding factors, histone modifications, and specialized histones (Table 1). Most binding maps were obtained in the Kc167 cell line, which provides a homogeneous cell population. Only the map of the variant histone H3.3 was derived from the S2 cell line, and the maps of eve and Prospero from *Drosophila* embryos. At present, this is the most extensive collection of genome-scale chromatin protein binding maps in a metazoan.

**Table 1:** Overview of binding profiles of chromatin components that were used in this study.

Mapped chromatin component	FlyBase protein accession	Transcription factor	Classical heterochromatin	Polycomb Group	Nuclear Envelope	Histone	Histone modification	HMG protein	Cofactor	Chromatin remodeling	Histone modifying enzyme	Data source	Cell line or tissue type	Mapping method
**bcd**	FBpp0081165	x										[44]	Kc167	DamID
**brm**	FBpp0075279									x		(a)	Kc167	DamID
**D1**	FBpp0081468							x				(a)	Kc167	DamID
**DSP1**	FBpp0088319			x				x				(a)	Kc167	DamID
**esc**	FBpp0079907			x								[21]	Kc167	DamID
**eve**	FBpp0087478	x										[26]	embryos	DamID
**Trl**	FBpp0089419	x										(a)	Kc167	ChIP
**gro**	FBpp0084337								x			(a)	Kc167	DamID
**H3K27me3**	-			x			x					[21]	Kc167	ChIP
**H3K4me3**	-						x					[21]	Kc167	DamID
**His1**	FBpp0085248					x						(a)	Kc167	DamID
**His3.3A**	FBpp0078649					x				x		[27]	S2	ChIP
**HP1**	FBpp0079251		x									[24]	Kc167	DamID
**Lhr**	FBpp0086073		x									[24]	Kc167	DamID
**HP4**	FBpp0076480		x									[24]	Kc167	DamID
**HP5**	FBpp0073349		x									[24]	Kc167	DamID
**HP6**	FBpp0077134		x									[24]	Kc167	DamID
**Jra**	FBpp0087499	x										[44]	Kc167	DamID
**Lam**	FBpp0078733											[22]	Kc167	DamID
**Max**	FBpp0074785	x										[44]	Kc167	DamID
**MBD-like**	FBpp0081547									x		(a)	Kc167	DamID
**Mnt**	FBpp0070554	x										(a)	Kc167	DamID
**Pc**	FBpp0078059			x								[21]	Kc167	DamID
**Prospero**	FBpp0088660	x										[25]	embryos	DamID
**Sce**	FBpp0084614			x								[21]	Kc167	DamID
**Sin3A**	FBpp0087003								x			(a)	Kc167	DamID
**Sir2**	FBpp0080015								x		x	(a)	Kc167	DamID
**SuUR**	FBpp0075933		x									[28]	Kc167	DamID
**Su(var)3-7**	FBpp0082204		x									(a)	Kc167	DamID
**Su(var)3-9**	FBpp0082583		x								x	[24]	Kc167	DamID

(a) This study

### Detection and Visualization of Clustering in Protein Binding Maps

The definition of a multi-gene chromatin domain is not trivial. Intuitively, it might be defined as a set of adjacent genes that are all bound by a chromatin protein X. However, it is conceivable that one or more genes loop out from a domain and do not bind X. In this case, the domain would consist of two or more sub-domains, and it is not obvious whether one should regard it as a single larger domain or as multiple smaller domains. Both views may in fact be correct; for example, the larger domain may determine the overall expression pattern of the included genes, while the sub-domains may act as separate fine-tuning units, and the intervening gene(s) may separate the units. This is just one theoretical example of a possible configuration; many different types of domain structures may exist [2],[3],[16],[17].

To obviate the need for detailed models, we took an unbiased statistical approach. We defined chromatin domains as regions of *local* enrichment in occupancy by a specific chromatin component over multiple adjacent genes. We require that this local enrichment is *statistically significant*, i.e., it must not be explainable by random fluctuations. Practically, this means that this local enrichment should not be observed when the order of genes in the genome is randomly permuted. To detect and visualize regions of local enrichment in our protein binding maps, we modified and extended a previously reported sliding window method [15] (see Methods). For each window of *w* consecutive genes, we tested whether the distribution of protein occupancy values differs from what is expected by chance, by comparing it to a null model in which the linear order of genes in the genome is randomly permuted. For each possible window position along each chromosome arm, and each possible window size, we accordingly computed a P-value representing the probability of observing the same or a larger degree of linear clustering by chance. Note that because all possible window sizes are analyzed, this approach allows for the identification of hierarchical structures of domains within domains. We emphasize that this approach does not require any pre-defined threshold for the level of protein occupancy, which would be arbitrary in the absence of objective criteria for choosing such a threshold.

**Figure 1:**
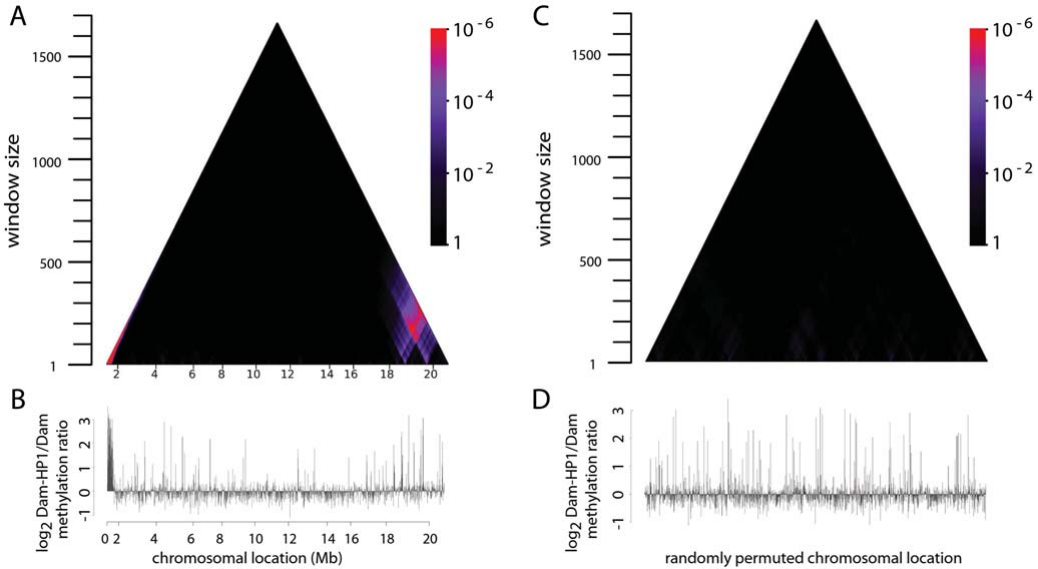
Visualization of chromatin domains by “domainograms”. To visualize local enrichment of a chromatin component, we calculate a probability score for the enrichment in a window of *w* neighboring genes under a null model in which all genes are randomly permuted. This calculation is done for all possible windows, ranging in size from a single gene to all genes on an entire chromosome arm, and for all possible window positions. A color scale (ranging from black for non-significant scores close to 1, to red for highly significant scores <10^−6^, see color scalebar) is used to visualize the probability scores in a triangular graph, which we term “domainogram”. Horizontally, each score is plotted at the chromosomal position of the center of the window, and vertically the windows are ordered by size. Thus, we obtain an intuitive visualization of local enrichments at all possible scales. See Methods for a detailed description. A) Domainogram of HP1 binding on the right arm of chromosome 2. B) Genomic map of HP1 binding used to generate the domainogram. C–D) domainogram plot and corresponding binding map after random permutation of the HP1 binding values along the genome. Genomic locations (Mb) are indicated below each graph in A–B.

To visualize the P-values that quantify the local enrichment of protein occupancy in multi-gene regions at all possible spatial scales for each chromosome, we use a triangular graph we call “domainogram”, in which window position is indicated on the horizontal axis, window size on the vertical axis, and P-value by a color scale. Fig. 1A shows a domainogram of the binding of Heterochromatin Protein 1 (HP1) on chromosome arm 2R. This graph reveals that a few large chromosomal regions are significantly enriched for HP1 binding (bright purple and red colors). The pericentromeric region shows strong enrichment of HP1, consistent with previous reports [29],[30]. In addition, a telomere-proximal region of highly significant enrichment is identified that was not previously known. Interestingly, this region displays a nested organization: two smaller regions of enrichment at ∼18 and 20 Mb together are part of a substantially larger region. No enrichments are seen after random permutation of the gene order (Fig. 1C,D), underscoring that our statistical criterion for spatial clustering is valid.

### Non-Random Local Enrichments Are Abundant and Can Be Dynamic

**Figure 2:**
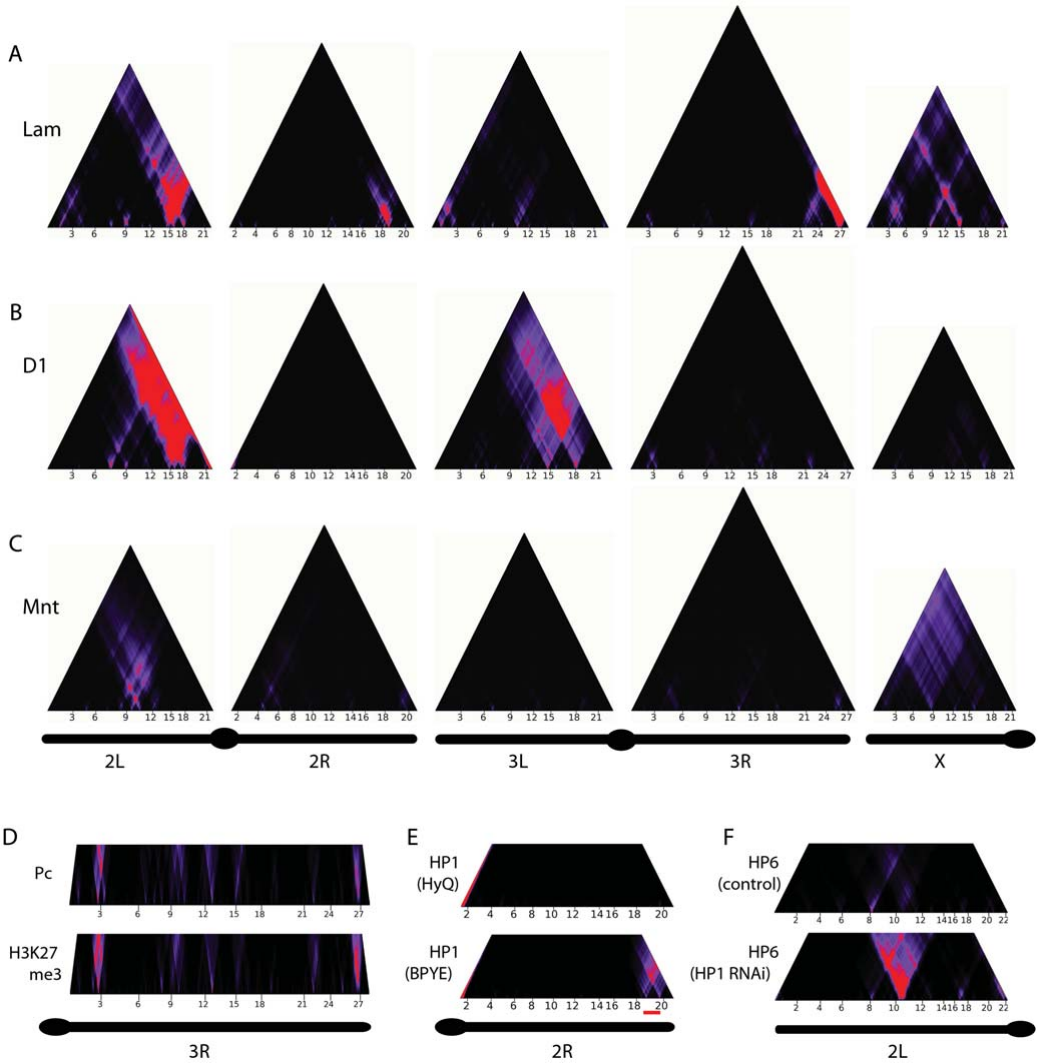
Genome-wide domainograms reveal non-random local enrichment of chromatin components. (A–C) domainograms for Lamin (A), D1 (B) and Mnt (C) along all major chromosome arms. Simple and nested patterns of local enrichment are visible. D) Domainogram comparison for Polycomb (mapped by DamID [21]) and H3K27me3 (mapped by ChIP [21]) on chromosome 3R. E) HP1 distribution on chromosome 2R in Kc167 cells grown in serum-containing (BPYE) and serum-free (HyQ) medium. A strong telomere-proximal region of enrichment is only observed in BPYE medium (indicated by the red bar). Data from BPYE medium is the same as in Fig. 1A–B. F) Domainograms of chromosome 2L for HP6 binding after RNAi of its binding partner HP1 and after a control RNAi (data from [24]). In D–F, only the bottom parts of the domainogram triangles are shown.

We systematically generated domainograms for all proteins in the compendium (Fig. 2 and Supplementary Fig. S1). Strikingly, nearly all proteins exhibit non-random enrichment at multiple sites in the genome. In some cases, such as for Lamin (Lam; Fig. 2A) and Polycomb (Pc; Fig. 2D) this is consistent with previously reported evidence for clustering of target genes [21],[22],[31]. For many other proteins, such as the HMG protein D1 and the transcription factor Mnt (Fig. 2B and C), the non-random genomic distribution has not been reported before. In several instances, the patterns of enrichment suggest a nested architecture, with larger domains subdivided into two or more smaller regions of enrichment (e.g., Fig. 2A–C). More complex enrichment patterns, sometimes covering a substantial part of a chromosome arm, can also be seen (e.g., D1 on chromosome 2L, Fig. 2B). Taken together, these results indicate that most chromatin components are highly non-randomly distributed along the *Drosophila* genome.

Most of the maps used for this analysis were obtained using cDNA arrays to detect protein binding. This means that genes are the units of mapping, and only protein binding at or in the flanking regions (about 1–2 kb on either side) of genes is detected [32]. To test whether this restriction might affect the identification of regions of enrichment, we also constructed domainograms of high-resolution tiling array DamID data of HP1. Comparison showed that cDNA array data yielded essentially the same enrichment patterns as tiling array data, although the latter provide a more fine-grained view (Supplementary Fig. S2). To rule out the possibility that the observed patterns of enrichment are the result of an experimental bias of the DamID technique, we compared DamID data for Pc with ChIP data for H3K27me3, the histone modification that forms the primary docking site for Pc [33] (Fig. 2D). Reassuringly, the domainograms are very similar.

We were surprised to find that 9 out of 29 proteins displayed moderate but significant enrichment along the entire X chromosome (note the purple or red colors in the top parts of the X chromosome domainograms in Fig. 2C and Supplementary Fig. S1). For histone H3.3 in male S2 cells this was previously reported and attributed to the dosage compensation mechanism [27], which ensures ∼2-fold increased expression of most genes on the single male X chromosome [34],[35]. The global X-enrichment of several other proteins (Bicoid, brahma, eve, Groucho, HP1, HP6, MBD-like, Mnt, Trl) in female Kc167 cells is surprising, but may be linked to the observation that X-linked genes in females also display slightly but significantly enhanced gene expression levels compared to autosomal genes [36].

To assess whether domains of enrichment are stable or dynamic entities, we compared HP1 binding patterns in Kc167 cells under two different culturing conditions, *viz*. medium with serum (BPYE) and without serum (HyQ). While some HP1 domains (e.g., in pericentric regions) remain constant under these two conditions, other domains appear to be dynamic (Fig. 2E and Supplementary Fig. S1). For example, the large telomere-proximal region of enrichment on chromosome 2R is only observed when the cells are grown in BPYE, and is completely absent in HyQ (Fig. 2E). This indicates that this region on 2R consists of a large cluster of conditional HP1 target genes that bind HP1 simultaneously upon an (yet unknown) intracellular signaling event that is triggered by serum. We have also studied the dynamics of chromatin domains formed by the protein HP6 by interfering with its interaction partner HP1 (Fig. 2F; data from ref. [24]). After knock-down of HP1, the formation of a prominent chromatin domain of HP6 binding is observed around position ∼10 Mb on chromosome 2L, a region that is also enriched in binding of Mnt (Fig. 2C) and several other proteins (see below). These results show that external signals or perturbation of chromatin complex composition can influence the formation of chromatin domains.

### Definition of Discrete Domains of Enrichment

While the domainograms are useful for visualizing regions of local spatial enrichment, they do not provide precise domain boundaries, as would be desirable for subsequent functional analyses (see below). To this end, we developed a dynamic programming algorithm that for each protein identifies the optimal genomic partitioning into discrete domains. To capture potentially nested domain structures, we performed this procedure iteratively using a maximum domain size constraint, and combined results for all possible values of this maximum domain size. As a result, the domainogram is simplified to a set of partially overlapping discrete domains of enrichment. For a detailed description of our algorithm, see Methods. We refer to the discrete domains identified by the partitioning algorithm as Blocks of Regulators In Chromosomal Kontext (BRICKs). We note that whereas some chromatin domains may be discrete in reality, others may have less sharply defined borders. In the latter case the discretization into BRICKs represents an oversimplification for practical purposes.

**Figure 3:**
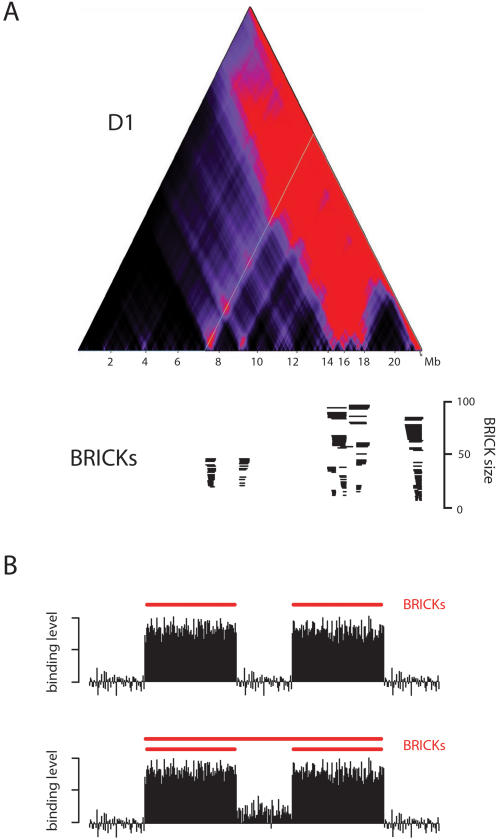
Identification of the most probable locations of discrete chromatin domains. An algorithm based on dynamic programming (see Methods) was used to identify the most probable partitioning into discrete domains of local enrichment (BRICKs). A) Top panel: domainogram of D1 on chromosome arm 2L. Bottom panel: corresponding locations of identified BRICKs (up to a BRICK size of 100). Nested BRICK structures can be identified, in which large BRICKs overlap with smaller BRICKs. They are visualized as a stack of BRICKs, and are all used for subsequent functional analyses. B) Simplified cartoon illustrating that the BRICK detection algorithm only combines two smaller BRICKs into one larger BRICK if the protein binding values between the two smaller BRICKs are significantly elevated above background. Thus, higher-level BRICKs are not just a trivial consequence of two smaller BRICKS being in close proximity of one another.

Fig. 3A shows the BRICKs identified for HMG protein D1 on chromosome arm 2L. When tested on simulated data that consist of various discrete domains placed in a noisy background, our algorithm identifies most domains correctly, with a low false-positive rate (Supplementary Fig. S3). Parameters were chosen such that for randomly permuted datasets the algorithm discovers ∼40 times fewer discrete domains than in the actual biological binding maps, i.e., the estimated false discovery rate (FDR) is ∼2.5%. Importantly, our algorithm was designed to discover the *intrinsic* size of the binding domains: a larger region containing two or more smaller BRICKs will only be parsed as a BRICK itself if increased binding also occurs in the regions in between the smaller BRICKs (Fig. 3B). Therefore, the nested domain structure that can be observed between 14–18 Mb in Fig. 3A presumably reflects a complex chromatin domain structure. Consistently, in computer simulations of chromosomes with simple discrete domains, our algorithm typically does not find nested or overlapping BRICK patterns (Supplementary Fig. S3).

### BRICKs Reveal Combinatorial Relationships between Subsets of Chromatin Proteins

**Figure 4:**
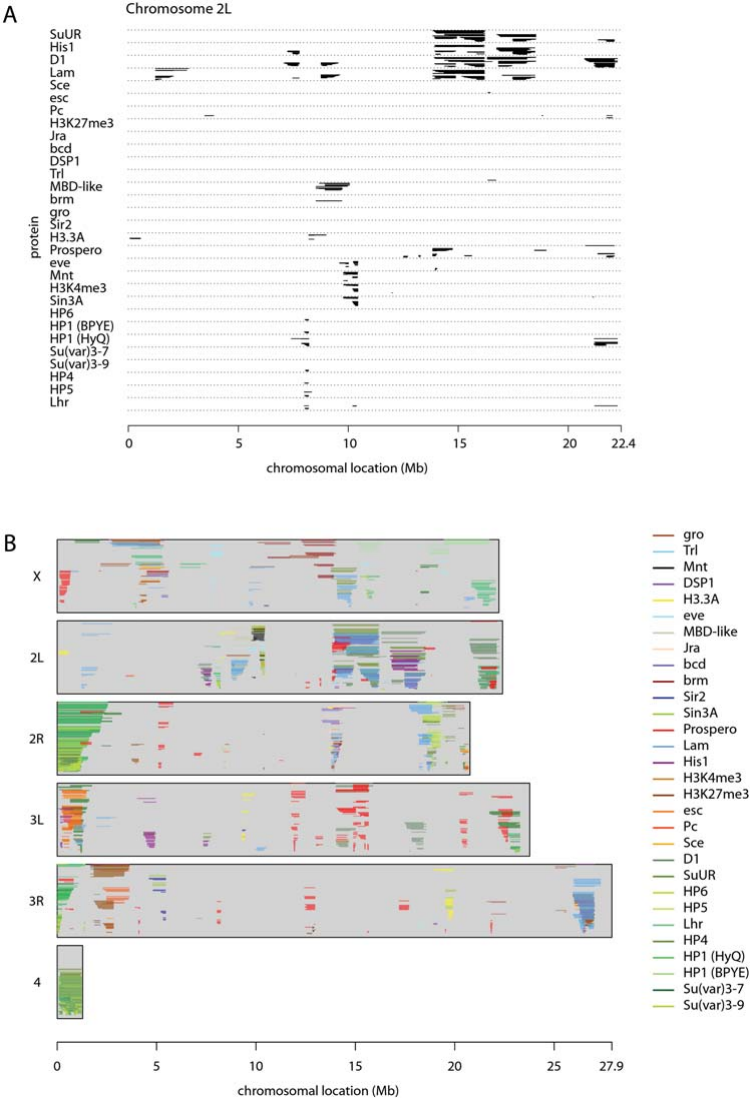
BRICK locations for all tested proteins. A) BRICKs on chromosome arm 2L. BRICKs smaller than 100 probed genes are shown for all analyzed proteins. The proteins are ordered by hierarchical clustering, with proteins that have the strongest overlapping domains closest together in the figure. B) Combined overview of the BRICKs for all proteins on all chromosome arms. BRICKs of different proteins are color-coded as indicated. Vertical position corresponds to the number of genes contained in the BRICK. Note that a substantial part of the *Drosophila* genome (∼50%) is covered by at least one BRICK.

To compare the spatial binding patterns of the 29 tested proteins and histone marks, we used a visual representation in which their respective BRICKs are stacked, providing a compact simultaneous view of their chromosomal domain structure (Fig. 4A and Supplementary Fig. S4). This revealed that several proteins have strongly overlapping BRICKs, suggesting that these proteins may act together to form a distinct chromatin domain. As expected, heterochromatin components HP1, Su(var)3-9, HP3/Lhr, HP4, HP5 and HP6 colocalize in BRICKS in pericentric regions (Supplementary Fig. S4) and can also be seen to form a small consistent domain at position ∼8 Mb on chromosome 2L (Fig. 4A). Likewise, the BRICK structures of the Polycomb Group complex components Pc, Sce, esc and H3K27me3 are highly similar. Other combinations of proteins are more surprising. For example, the BRICKs for Mnt, H3K4me2, Sin3A, and eve overlap strongly on chromosome 2L around ∼10 Mb (Fig. 4A). BRICKs of Lamin, His1, D1, and SuUR also overlap, between ∼14 and ∼18 Mb on chromosome 2L. Some proteins can be part of different types of domains: In pericentric regions, D1 shares BRICKs with HP1 and other heterochromatin components, but at other sites D1 is found in various combinations with Lam, SuUR and His1 (Supplementary Fig. S4). Similarly, Sin3A forms different combinations with Sir2, H3K4me2, and Mnt (Fig. 4A and Supplementary Fig. S4), and also with H3.3 and eve (with the caveat that the latter profiles were not obtained in the Kc167 cell line). These results are suggestive of a combinatorial “chromatin code” that marks specific domains.

A merged overview of BRICKs in all chromosomes (Fig. 4B) reveals that a substantial part of the *Drosophila* genome is organized into chromatin domains. When BRICKs are limited to a maximum of 100 consecutive genes, 50% of the genome, corresponding to 54% of all genes, is covered by at least one BRICK. These results demonstrate a strikingly high degree of non-random organization of genes into chromatin domains.

### BRICKs Represent Functionally Relevant Genomic Domains

BRICKs typically show average protein binding log-ratios ranging from ∼0.4–3 (Supplementary Fig. S5), which corresponds to ∼1.3–8 fold enrichments of a chromatin component in each BRICK relative to the genome-wide median value. Even subtle modulations of protein-genome interactions may have biologically relevant effects on gene regulation, but functional evidence is required to confirm this. To directly address whether BRICKs represent chromatin domains of functional importance, we performed three different analyses.

**Figure 5:**
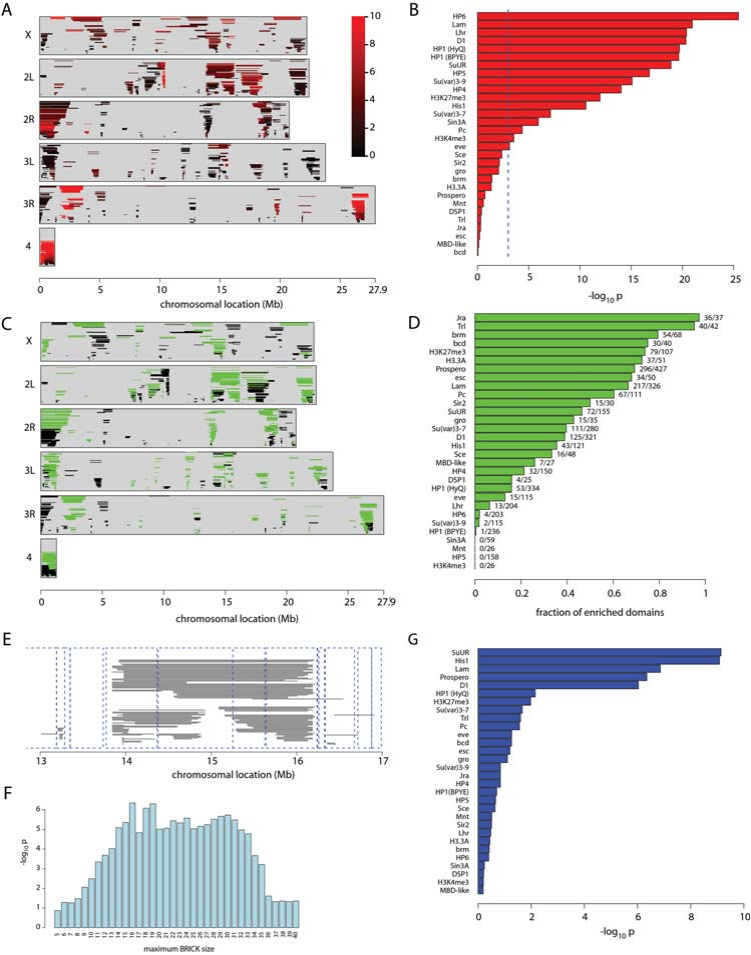
Evidence for functional relevance of BRICKs. A–B) Developmental coexpression of genes within BRICKs. A) Combined BRICKs of all proteins as in Fig. 4B, colored for the relative degree of developmental coregulation of the genes within each BRICK (average pairwise correlation between all the genes in the domain). To be able to compare BRICKs of different sizes, we normalized the average pairwise correlation to a z-score by dividing by the standard deviation of 1000 average pairwise correlations of a random subset of *n* genes (see Text S1). B) Statistical significance of coregulation of genes within BRICKs, for each chromatin protein. For each BRICK a quantile score was determined, representing the rank of the coregulation in the BRICK, compared to the set of all equally-sized windows. The *P*-value was calculated using a Kolmogorov-Smirnov test for deviation from a uniform distribution, representing the null hypothesis that BRICKs do not show more coregulation than non-BRICK windows (see Text S1 for details). The dotted line indicates the significance threshold (p<0.001). C–D) Shared functions of genes within BRICKs. C) BRICKs that are significantly enriched for at least one GO category at an FDR cut-off of 1% are marked in green (see Methods). D) The fraction of GO-enriched BRICKs for every chromatin protein. Next to each bar are the absolute numbers of GO-enriched and total BRICKs, respectively. E–G). Reduced numbers of synteny breakpoints within BRICKs. E) Part of chromosome arm 2L, showing positions of synteny breakpoints (dotted blue vertical lines) relative to BRICKs (black horizontal lines). Note that breakpoints tend to be located just outside BRICKs. F) Statistical significance of exclusion of synteny breakpoints from BRICKs formed by Prospero. Combined BRICKs for all proteins, up to the indicated BRICK size, were tested for exclusion of synteny breaks using a hypergeometric test. G) Statistical significance of exclusion of synteny breaks from BRICKs separated by chromatin protein. The *P*-values are the smallest values taken from plots as in F) (see Supplementary Fig. S9).

First, we hypothesized that genes may be packaged together in a BRICK to facilitate their synchronized expression during development. To test this, we determined the degree of developmental coexpression of genes within each BRICK, using a previously published *Drosophila* gene expression dataset [37]. Fig. 5A illustrates that a large fraction of the BRICKs indeed show substantial coregulation. Because neighboring genes are often coregulated [13],[37], we asked specifically whether genes within BRICKs display a higher degree of coexpression than genes in size-matched control windows located outside BRICKs. Statistical analysis of these data (Fig. 5B, see Text S1 and Supplementary Fig. S6) demonstrates that for about half of the investigated chromatin proteins the degree of coregulation is significantly higher within BRICKs than in control windows. This indicates that many BRICKs may be important for the developmental synchronization of gene sets. We note that this analysis is based on the assumption that chromatin domains remain unaltered between the cells in which the protein binding patterns were mapped (mostly Kc167 cells) and the developmental stages for which expression data was obtained (six different stages ranging from early embryos to adult flies [37]). While several reports indicate that some chromatin domains indeed are very similar in different cell types, tissues, and developmental stages [21],[22],[38], other domains are more plastic (e.g., Fig. 2E and [22]). Our coexpression analysis does not take into account such potential dynamics in domain structure, and therefore may be expected to underestimate the correlation between BRICK organization and coordinated gene expression.

Second, we asked whether genes within each BRICK have common functions. To this end, we tested for enrichment of specific Gene Ontology (GO) categories [39] within each BRICK (see Methods). Fig. 5C shows that, at an estimated FDR of 1% (Supplementary Fig. S7 and Text S1), roughly half of all BRICKs are enriched for one or more GO categories. This number is significantly higher than what is expected by chance, even if the known genomic clustering of GO categories [7] is taken into account (P = 0.017, based on 1,000 genome-wide circular permutations of the association between genes and GO categories). A striking example is a BRICK defined by the protein Prospero (Supplementary Fig. S8); in this BRICK many genes encode transcription regulators that are implicated in the Notch pathway. In total, we find 150 GO categories enriched in one or more BRICKs (data not shown). Fig. 5D summarizes the fraction of GO-enriched BRICKs for all proteins separately. In conclusion, the observation that BRICKs are frequently enriched for genes with related functions argues that they are likely to serve as functional modules. Comparison between Figures 5A and C shows that BRICKs enriched for GO annotation are often not enriched for coexpression, and vice versa.

Third, we reasoned that if BRICKs are functionally important, chromosomal rearrangements that disrupt the BRICK structure should be subject to negative selection during evolution. To test this, we analyzed the positions of synteny breakpoints in the genome of *D. melanogaster* relative to *D. pseudoobscura* [40]. These two species diverged about 25–50 million years ago [41]–[43]. Indeed, we find that synteny breakpoints are often located adjacent to BRICKs, rather than within BRICKs (Fig. 5E). Statistical analysis shows that BRICKS defined by His1, Prospero, Lamin, SuUR and D1, with sizes up to 40 probed genes, have significantly fewer synteny breaks (∼67% reduction) than expected based on the distribution of synteny breaks in the genome (Fig. 5F–G and Supplementary Fig. S9). Larger BRICKs typically do not show this reduction, possibly because their integrity as a single domain is less important, or because they cannot be preserved at the high overall rate of synteny breaks (on average one breakpoint per 15 genes, median is 8). While we cannot strictly rule out that syntenic breaks and chromatin domain boundaries have a common mechanistic origin, the apparent evolutionary selection against the break-up of chromatin domains suggests that many of them are functionally important.

Together, these three lines of evidence support the functional importance of BRICKs in the *Drosophila* genome.

### General Sequence Properties of BRICKs

**Figure 6:**
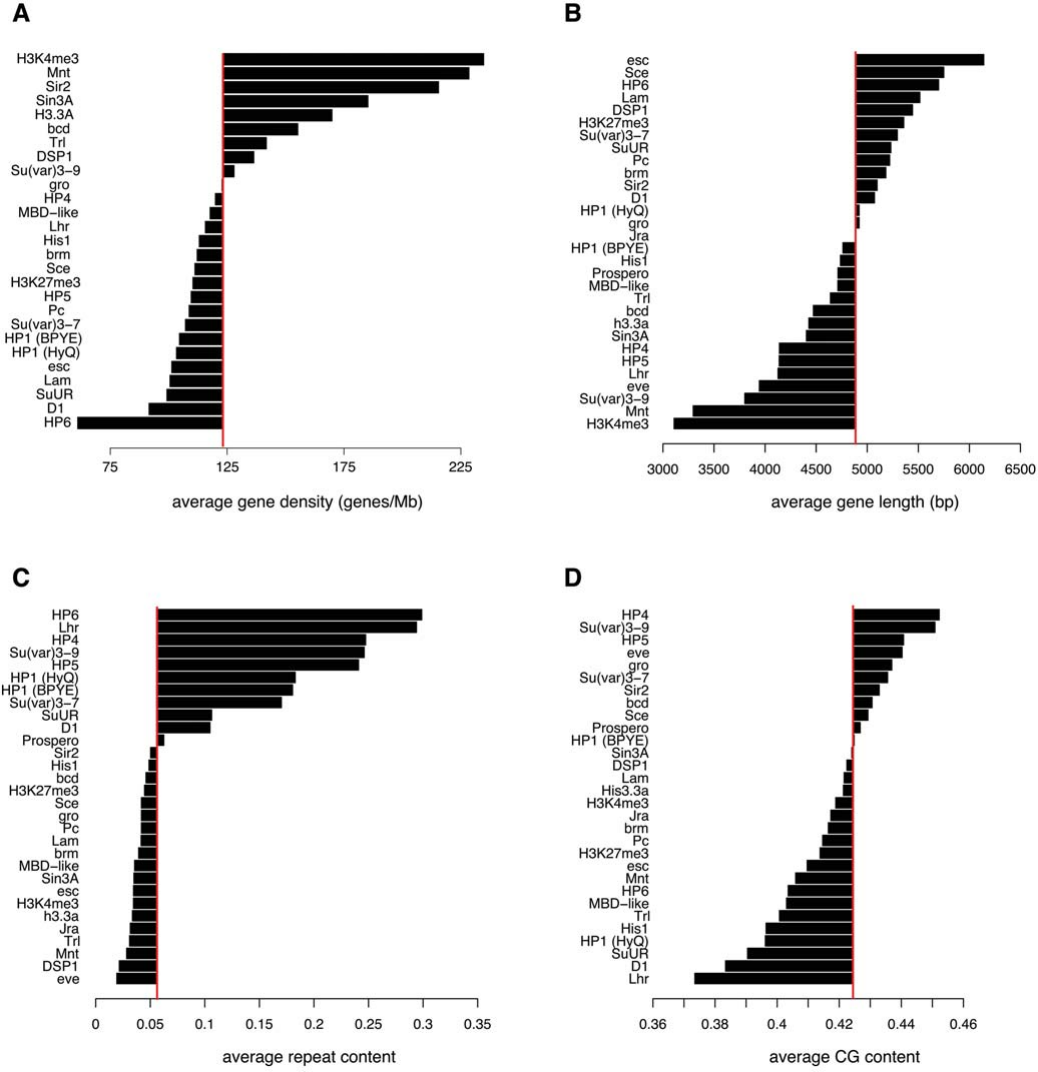
Sequence properties of BRICKs. For each type of BRICK (defined by a single chromatin component) the average value is plotted for A) gene density; B) gene length; C) fraction of repetitive (i.e., non-unique) sequence; D) G/C content. All values are plotted as deviations from the genome-wide average (red vertical lines).

Finally, we asked whether BRICKs represent regions with specific general sequence properties. First, we tested whether BRICKs are regions of unusual gene density. For the set of BRICKs of each protein we calculated the average gene density, and compared it with the genome-wide average gene density (Fig. 6A). This analysis shows that different sets of BRICKs vary substantially in gene spacing. For example, consistent with previous observations [22], genes within Lam BRICKs are relatively widely spaced. The same is true for BRICKs of other heterochromatin proteins, such as SuUR, esc and HP1. By contrast, genes within BRICKs of H3K4me3, Mnt, and Sir2 have very short intergenic regions. Also the lengths of genes within BRICKs can vary between proteins (Fig. 6B). In BRICKs associated with inactive chromatin (esc, Sce, Lam) genes tend to be longer than in BRICKs of active chromatin (H3K4me3, Mnt). Analysis of repeat content (Fig. 6C) showed that BRICKs formed by classical heterochromatin proteins such as HP1, Su(var)3-9 and HP1-associated proteins [24] are more repeat-rich than other BRICKs, which is consistent with previous analyses [29],[38]. BRICKs defined by individual proteins show only minor variation in G/C content (Fig. 6D). It is important to note that the combined BRICKs for all proteins do not show a systematic bias related to gene density, gene size, repeat density, or G/C content. Therefore it is unlikely that their detection is a systematic artifact of variation in any of these parameters along the genome.

## Discussion

The results presented here indicate that about half of the *Drosophila* genome is organized into large chromatin domains, most of which are functionally relevant. This estimate of the coverage of the genome by domains is likely to be an underestimate for three reasons. First, because the BRICK segmentation algorithm is computationally intensive, we restricted the BRICK sizes to a maximum of 100 genes. The domainograms however indicate that substantial non-random clustering also occurs above this limit. Second, even though our compendium of binding maps includes a wide range of known protein complexes, many other proteins must be mapped for a complete view. Third, we provided evidence that at least for some chromatin proteins the domain structure may depend on the cellular state. We predict therefore that maps of protein binding in various cell types will reveal additional, cell-type specific BRICKs. Taken these considerations into account, our estimate that approximately half of the fly genome is organized in chromatin domains is conservative.

Previous analyses of genome-wide expression data have revealed that there are domains of similarly expressed genes in the genome of *Drosophila* [Spellman, 2002, 12144710; Boutanev, 2002, 12478293; Stolc, 2004, 15499012]. Spellman and Rubin have shown that ∼20% of the genome can be found in coregulated domains ranging in size between 10 and 30 genes, with a median of 13. The BRICKs range in size between 2 and 100 with a median of 26. However, we stress that due to the very different nature of the methods that were employed in both studies this comparison should be interpreted with caution.

The domainograms and BRICK patterns suggest that chromatin domains can have a complex, nested structure. It is tempting to speculate that looping interactions take place in such nested regions. It is noteworthy that transcription factors such as Trl, bcd, and Jra also exhibit spatial clustering. These factors do not spread along the chromatin fiber but instead have focal binding sites [44]. BRICKs of transcription factors must therefore be interpreted as non-random clusters of focal binding sites. Genes in BRICKs defined by transcription factors generally do not show simple coexpression but tend to have common functions (Fig. 5B,D). This is reminiscent of the mammalian β-globin locus, in which functionally related genes are not coexpressed but instead are transcribed in a temporally defined order. Several transcription factors have multiple binding sites in the β-globin locus [45], and looping interactions play an important role [19]. We therefore speculate that some of the transcription factor BRICKs may be similar in structure to the β-globin locus. Our BRICK database (provided in GFF file format as Dataset S2) provides a rational starting point for the selection of loci to probe for looping interactions using the 3C/4C/5C technologies [46].

The surprisingly widespread occurrence of chromatin domains has two major implications. First, chromatin domains provide a plausible explanation for earlier observations that neighboring genes in eukaryotic genomes are often co-regulated [13],[37]. Our results suggest that chromatin domains may at least be partially responsible for the synchronized expression of neighboring genes, although it cannot be ruled out that in some instances the clustering of a chromatin mark may be the consequence rather than the cause of this synchronized expression. Second, our data suggest that chromatin domains impose considerable constraints on genome evolution. Most likely, this is due to negative selection of genome rearrangements that disrupt the integrity of chromatin domains, but it is also possible that chromatin domains stabilize the chromatin fiber and thereby physically prevent chromosome rearrangements. In summary, the widespread chromatin domain organization provides new clues towards the understanding of the mechanisms of transcription regulation as well as genome structure and evolution.

## Methods

### DamID and ChIP-on-Chip Data

Table 1 summarizes the protein binding maps that we used. Published DamID and ChIP-on-chip profiles were taken from refs [21], [22], [24]–[28],[44]. In addition, we generated new DamID profiles for brm, Trl (GAGA factor), gro, Mnt, Sin3A and Sir2 using previously reported Dam-fusion expression vectors [44], [47]–[49], and for full-length D1, DSP1, His1, MBD-like, and Su(var)3-7 using newly constructed Dam-fusion vectors. These new profiles were generated in Kc167 grown in serum-containing medium as described [44]. The DamID profile of HP1 in Kc167 cells grown in serum-free Hyclone Insect-Xpress medium (“HyQ”) was not previously published but was generated in parallel with our already published profile of HP1 from cells in serum-containing medium [24], allowing for direct comparison. Plasmid sequences are available at http://research.nki.nl/vansteensellab. DamID experiments were performed as described previously [50]. Binding profiles represent the average of triplicate experiments, with one experiment in the reversed dye orientation. Log_2_ ratios were averaged across replicates. The raw data can be accessed via the Gene Expression Omnibus under accession number GSE10219; the combined binding data is also provided as Dataset S1, and the set of BRICKs is supplied in GFF format as Dataset S2. All data were generated in Kc167 cells, except for the maps of His3.3A, eve and Prospero. His3.3A data are from the S2 cell line [27]; Prospero [25] and eve [26] data are from stage 10–11 and stage 17 embryos, respectively.

Except for the eve and Prospero maps, all data were generated using 12k cDNA arrays. Each cDNA probe detects the binding at or in the vicinity (∼1–2 kb) of a gene [32]. Thus, genes are the units of our analysis. To ensure that each cDNA probe constitutes an independent datapoint, overlapping cDNA probes were removed, using the following rules: 1) if a probe overlapped with multiple other mutually non-overlapping probes we removed the former probe from the dataset, 2) if two probes overlapped more than 20%, the smaller of the two probes was removed.

Binding data of eve and Prospero were generated with genome-wide tiling arrays [25]. To allow for direct comparison with the cDNA array based data, we resampled the tiling array data, so that we had one datapoint per gene. For this we used the gene annotation from release 4.3 of the Flybase genome annotation (http://www.flybase.net). For every gene in the genome we calculated the average of all the probes encompassed by that gene. As with the cDNA data, when two genes overlapped more than 20%, the smallest gene was removed, with the exception of genes that overlapped with multiple non-overlapping genes, in which case the gene overlapping with multiple genes was removed. After removal of overlapping genes we are left with 12,821 genes for which we have reliable eve and Prospero data.

For the comparison of cDNA data to high-resolution data, the HP1 tiling array data was not resampled to one datapoint per gene. For the comparison we used the left arm of chromosome 2, which contains >222k probes (1 probe per 100 bp) [29]. Since for large numbers of probes the domainogram analyses become computationally intensive, the tiling array data was averaged into equal-sized bins of 3 kb. These data were used as input to the algorithm.

### Computation of P-Values for Multi-Gene Windows

Because DamID and ChIP log-ratios for a specific protein are often not normally distributed (data not shown), we used a non-parametric approach to evaluate local enrichment. For each binding profile, probes are sorted in descending order according to their DamID or ChIP ratio and converted to single-gene quantile scores:
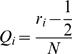
Here *N* equals the total number of probes and *r_i_* is the rank for probe *i* = 1,…,*N*. To integrate evidence for protein occupancy across multiple adjacent probes for each window (*i,w*) of width *w* ending at probe *i*, we compute a *multi-gene P-value*, *P_iw_*, from the single-probe quantile scores (*Q_i−w+1_*, … , *Q_i_*), with *i*≥*w*. We define *P_iw_* so as to have a uniform distribution on the interval [0,1] if all the *Q_i_* values, which are uniformly distributed by construction, are independent random variables. To this end we use a transformation according to R.A. Fisher [51]: Given the product statistic
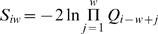
 can be computed using a χ^2^-distribution with *2w* degrees of freedom:
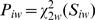
Note that for *w* = 1, we have *P_i1_ = Q_i_*. The *P_iw_* can be visualized simultaneously in a triangular diagram (“domainogram”) using an approach similar to Versteeg et al. [15]. Image files were created using custom Perl scripts (available upon request).

### Dynamic Programming Procedure for Defining BRICKs

To identify the most probable discrete domains of size >1 (BRICKs) from the *P_iw_* data structures, we used a dynamic programming algorithm [52]. We modified our scoring scheme so as to favor the “no-domains” segmentation consisting of only *w* = 1 windows by introducing a bias factor γ and defining:
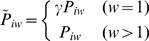



Each possible segmentation of the genome into non-overlapping windows corresponds to a path {(*i*(*k*), *w*(*k*))} through the *P_iw_* triangle, where *k* = 1,...,*K* runs over all *K* windows in the segmentation (*K*≤*N*). Here *i(k)* denotes the last gene in the *k*-th window, while *w(k)* denotes the length of the *k*-th window. The optimal segmentation minimizes an objective function equal to the product over all windows constituting the path:
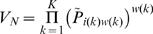
This segmentation can be determined using the recursion relation
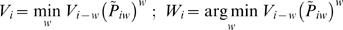
and the initial condition *V_0_*  =  1. Backtracking starting from *i*  =  *N* according to
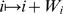
defines the optimal segmentation.

To identify the nested structure present in the domain organization, we perform the previously discussed computation, with restriction of the maximum window size (*w_max_*). This way the segmentation is restricted to smaller window sizes, which leads to the identification of smaller BRICKs. The analysis is iterated until the segmentation for all *w_max_*>1 has been determined. See Text S1 for more detailed information on the BRICKs algorithm.

### GO Enrichment Analysis

The Flybase Gene Ontology annotation version 1.92 was used to calculate the enrichment of GO categories. GO categories containing fewer than 5 genes were ignored. Enrichment of GO categories in each BRICK was determined using the cumulative hypergeometric distribution, accounting for multiple testing of all combinations of domains and GO categories. As both the BRICK structure and the GO dataset are hierarchically organized, we estimated the FDR using a Monte Carlo simulation in which all genes were randomly permuted while keeping the assignment to GO categories and BRICK structure intact. For each BRICK *d*, the *P*-value (cumulative hypergeometric distribution) for each GO category was determined, and the smallest of these was recorded as *P_d_.* The false discovery rate for each BRICK is then given by *FDR*(*P_d_*), where
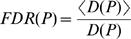



In the denominator, D(P) represents the number of BRICKs with a minimal p-value smaller than P, while the numerator represents an average of the same quantity over random permutations. Fig. S6 shows the distribution of the actual p-values belonging to the BRICKs for all proteins combined and the p-value distribution from 10,000 random genome permutations.

We have also performed this analysis for BRICK sets defined by individual proteins. In this analysis the FDR cut-off was based on 1000 randomizations. We have used this per protein calculation of the FDR cut-off to determine the number of enriched BRICKs shown in Fig. 5D.

A circular permutation test was performed to account for possible uneven distribution of GO category members across the genome. In this analysis we circularly permuted the genes along the BRICK set. Using the above mentioned *FDR*(*P_d_*) as a cut-off we determined the number of BRICKs (*BRICK_GO_*) that fell below this threshold. The distribution of *BRICK_GO_* of 1000 circular permutations is compared to the actual number of significantly enriched BRICKs to determine the *P*-value.

### Synteny Analysis

Release 4.3 of the *D. melanogaster* genome annotation (Berkeley Drosophila Genome Project) contains information on the start and end location of regions that are syntenic to genomic regions in *D. pseudoobsura*. These locations represent synteny breakpoints. Since it has been reported that the scaffolds from the dot chromosome (chromosome 4 in *D. melanogaster* and chromosome 5 in *D. pseudoobscura*) could not reliably ordered in *D. pseudoobscura* [40], we omitted chromosome 4 from our synteny calculations. On the other chromosome arms of *D. melanogaster*, the distribution of the synteny breakpoints is not significantly different from a uniform random distribution (Kolmogorov-Smirnov test, P = 0.6498; data not shown).

Synteny blocks spanning multiple genes sometimes contain insertions of a single gene from a different locus in *D. pseudoobsura*. In the genome annotation, these events are marked by two syntenic block entries. We decided that insertion of a single gene does not constitute a break in a synteny block, when it is embedded in a larger region of synteny. For the formation or break-up of chromatin domains, insertion of a single gene is likely a less deleterious event then an actual break in synteny.

Depletion of synteny breakpoints from BRICKs was determined as follows: Given the start and end position in a BRICK, we determined the genes that are encompassed by the BRICK. Since breaks in synteny almost exclusively occur in between genes, we counted the number of intergenic regions within all the BRICKs (*n*). Next we determined the number of synteny breakpoints within the BRICKs (*k*). Given that there are 955 synteny breakpoints (*K*) in the *D. melanogaster* genome and 14,351 intergenic regions (*N*), we can calculate a probability score using the cumulative hypergeometric distribution for *k* syntenic breakpoints in a BRICK containing *n* intergenic regions.

The median synteny block size is 8 genes, whereas some BRICKs are much larger (by definition up to 100 genes). Because of this partial discrepancy in scale, we performed the synteny analysis for subsets of BRICKs smaller than a given maximum size (BRICK size is the number of probed genes per BRICK). Plotting –log_10_(*P*-value) as a function of the maximum BRICK size visualizes the size-dependent depletion of synteny breakpoints from BRICKs (Fig. S8). Fig. 5G shows the *P*-values corresponding to the most significantly depleted BRICK size range for each protein.

## Supporting Information

Figure S1 Domainograms for all tested proteins on all major chromosome arms. Chromosome 4, which is only ∼1.2 Mb in size, is not shown. Color scheme is the same as in Figure 1.
(1.46 MB PDF)

Figure S2 Comparison of cDNA data with high-resolution tiling array data. Domainograms for high resolution tiling array DamID data (top) and cDNA array DamID data (bottom) for HP1 on chromosome 2L.
(0.81 MB PDF)

Figure S3 Domainogram and BRICK identification from synthetic data. Simulated data were generated to test the visualization and detection of chromatin domains. We created a virtual chromosome arm of 1200 genes, each associated with a quantile score (range 0–1) representing the ranked binding of a virtual protein. On this chromosome arm we placed seven domains consisting of 5–100 neighboring genes that were assigned quantile scores representing either “strong” (randomly selected quantile scores 0.99–1.00), “medium” (0.90–0.99) or “weak” (0.75–0.90) binding. The remainder of the genes was assigned a random value. A) Domainogram derived from an artificial dataset, and B) the corresponding simulated data. Yellow, orange and red rectangles denote the domains of weak, medium, and strong binding, respectively. C) Plot showing the performance of BRICK detection on 100 separate simulation runs. Horizontal lines denote the coordinates of identified BRICKs in each simulation run (vertical axis). Sensitivity of BRICK detection depends on the size and intensity of the domain, but identification of spurious domains or fusion of separate domains occurs very rarely.
(0.75 MB PDF)

Figure S4 BRICK plots showing the distribution of chromatin domains for all proteins on each chromosome arm. BRICKs <100 probed genes are shown. Each horizontal line depicts the position of a BRICK. For each protein, the relative vertical location of the lines represents the number of probed genes in a BRICK.
(0.38 MB PDF)

Figure S5 Enrichments of protein binding in BRICKs. Boxplots are shown of the average protein binding (DamID or ChIP) logratio for each BRICK size. Boxes show 25th–75th percentile, and the horizontal line inside each box indicates the median.
(0.16 MB PDF)

Figure S6 Empirical cumulative distribution plots for quantile scores for coregulation in domains. Figures show cumulative distribution of quantile scores of coregulation (see Text S1 for details on the calculation of quantile scores). Each figure represents the coregulation level for one protein as indicated. Horizontal axes represent quantile scores, vertical axes represent the relative cumulative level for a given quantile score. The dashed gray line represents the theoretical uniform distribution. In the top-left corner of each graph is indicated the p-value according to the KS-test, for deviation from a uniform distribution.
(0.94 MB PDF)

Figure S7 Empirical cumulative distribution of p-values for enrichment of GO categories. P-values of enrichment for GO categories were calculated using the cumulative hypergeometric distribution. Empirical distribution of the p-values in the domains is shown in black. The gray line denotes the empirical distribution of p-values from 10,000 randomized genomes. The red dashed line denotes the p-values for which the FDR is 0.01.
(0.27 MB PDF)

Figure S8 A Prospero chromatin domain is enriched for genes encoding transcription factors involved in Notch signaling. A) Bottom part of a domainogram of chromosome 3R for Prospero binding. Below the plot the corresponding BRICK structure is shown. B) Chromosomal map showing tiling array data with log2 binding ratios for Prospero (Choksi et al. Dev Cell. 2006 Dec;11(6):775-89) in a BRICK region. C) Genes located in the same region. Three major GO categories are indicated by different colors.
(1.07 MB PDF)

Figure S9 Synteny breakpoints are significantly depleted from BRICKs defined by some proteins. Depletion of synteny breakpoints from BRICKs is calculated using the cumulative hypergeometric distribution. For every protein, barplots show the p-value as a function of the maximum BRICK size. For a maximum BRICK size, all BRICKs up to that size are included.
(0.47 MB PDF)

Text S1 Additional information on computational methods.
(0.57 MB PDF)

Dataset S1 Genome-wide binding data for all analyzed chromatin components.
(1.72 MB ZIP)

Dataset S2 GFF file listing all BRICKs.
(0.39 MB TXT)
