# Supplementary material for: Global Chromatin Domain Organization of the Drosophila Genome
Source: PLoS Genet. 2008 Mar 28;4(3):e1000045. doi: 10.1371/journal.pgen.1000045 (PMC2274884; doi:10.1371/journal.pgen.1000045)
Supplement: Figure S1 — Domainograms for all tested proteins on all major chromosome arms. Chromosome 4, which is only ∼1.2 Mb in size, is not shown. Color scheme is the same as in Figure 1. (1.46 MB PDF) [file pgen.1000045.s001.pdf]

Figure S1

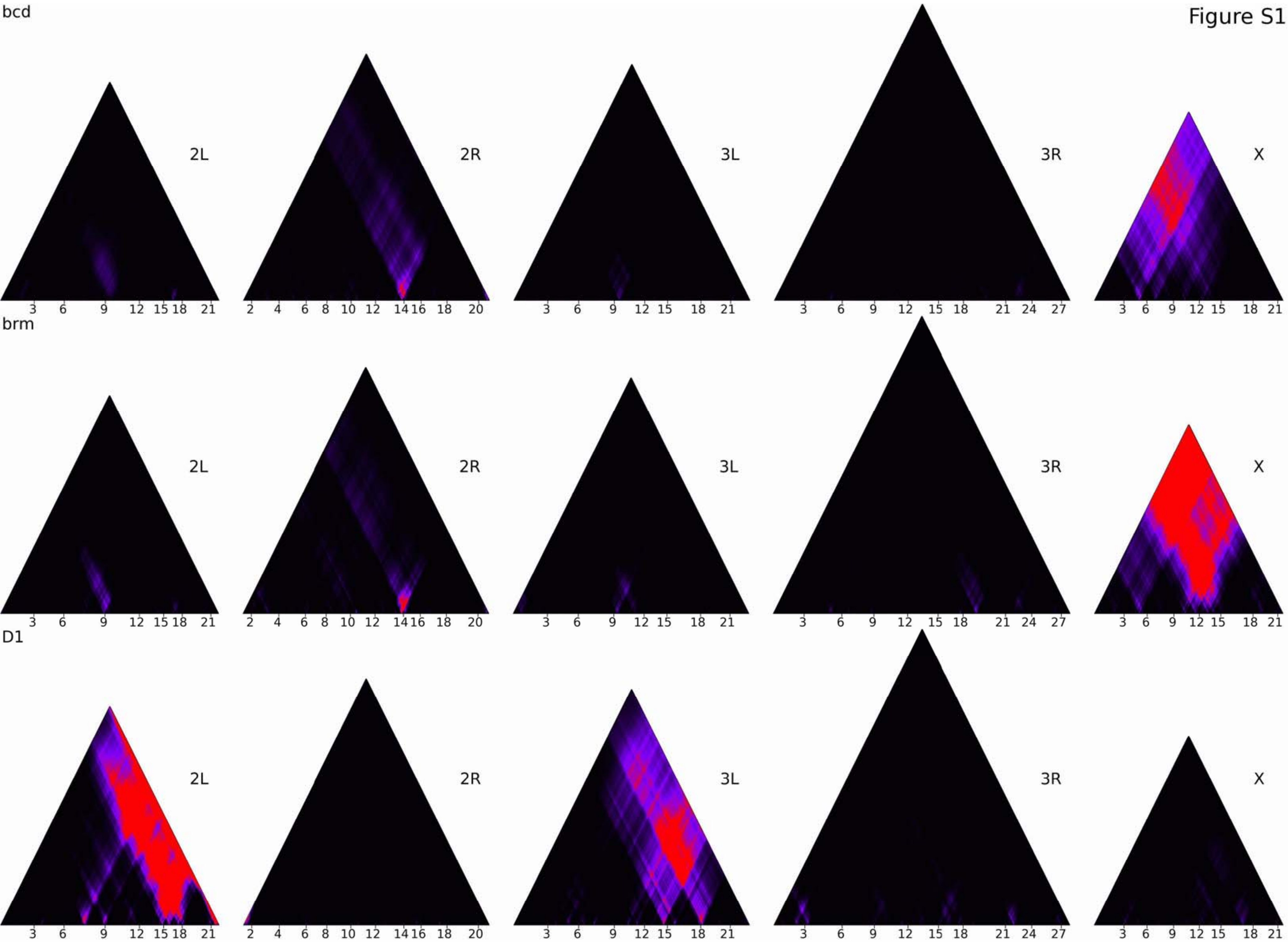

Figure S1

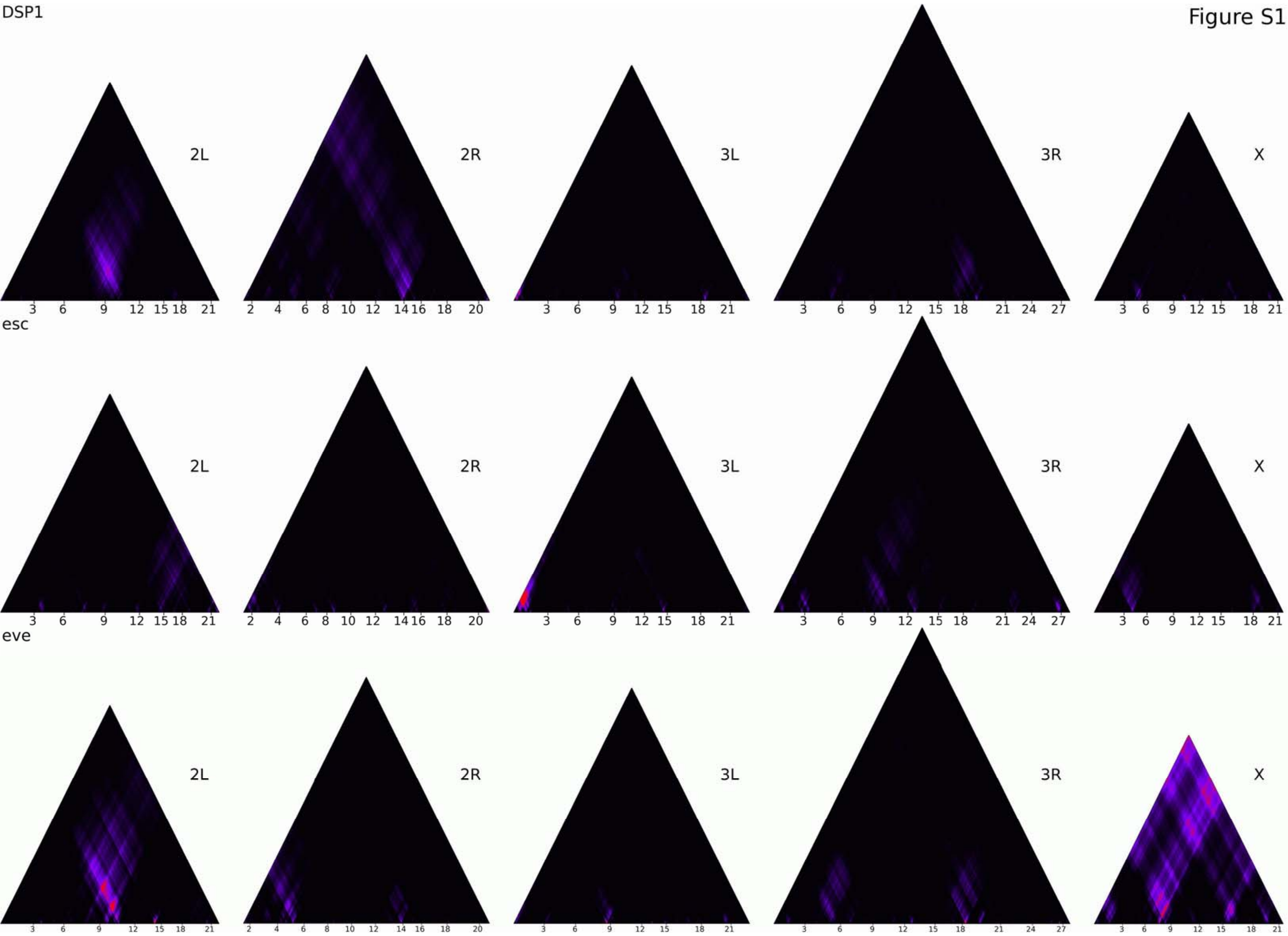

Figure S1

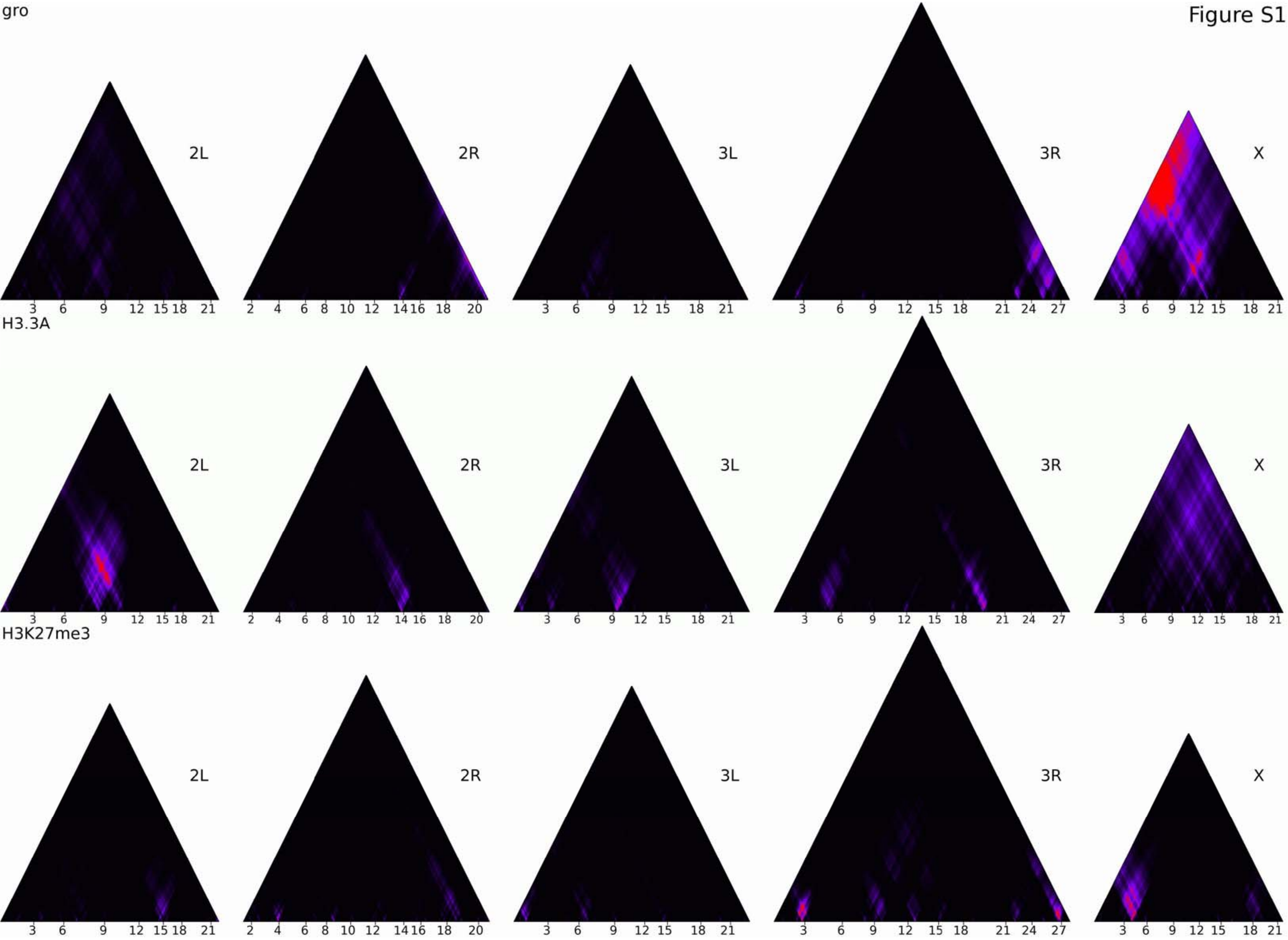

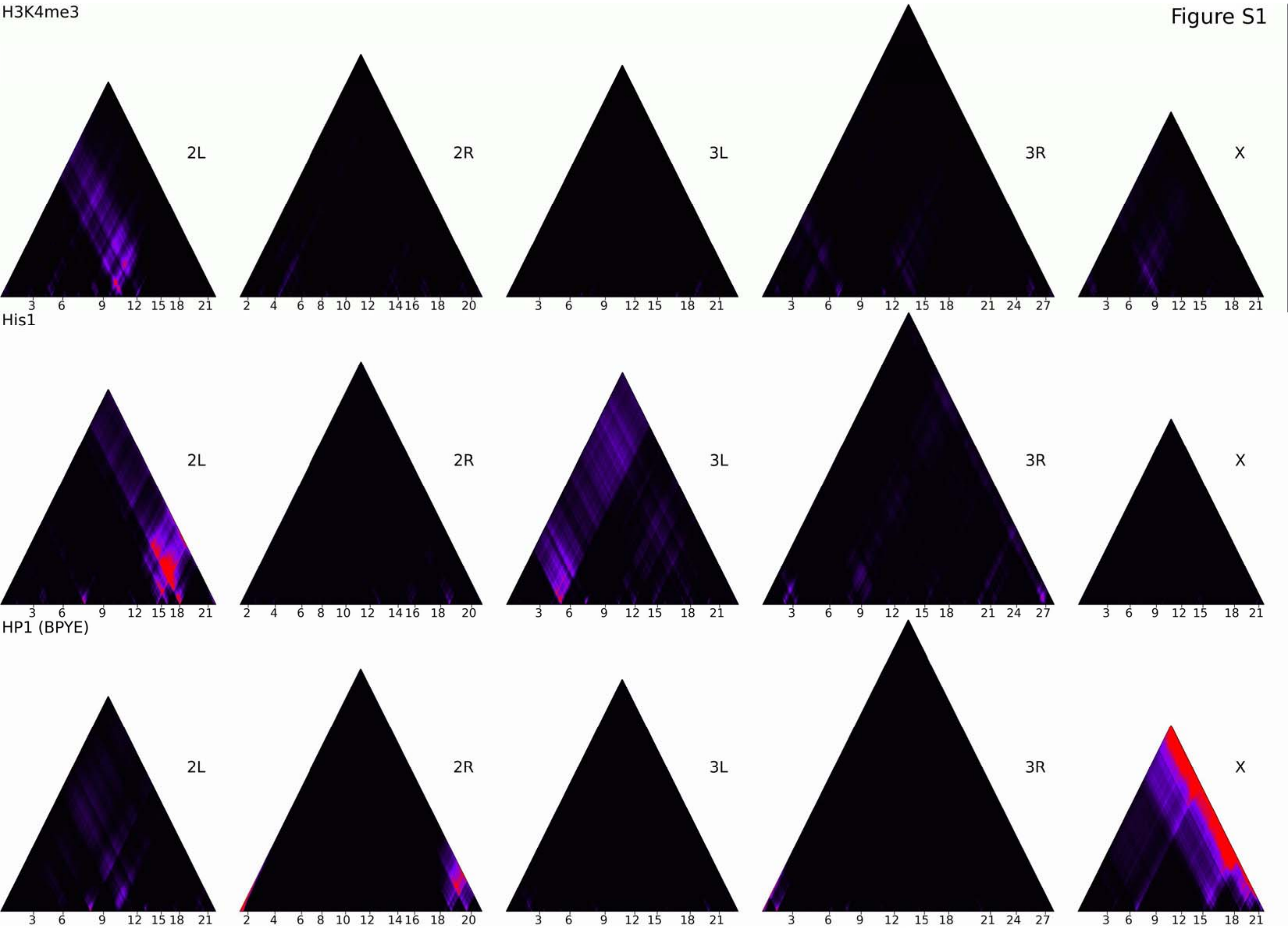
Figure S1
H3K4me3
2L
2R
3L
3R
X
3 6 9 12 15 18 21
2 4 6 8 10 12 14 16 18 20
3 6 9 12 15 18 21
3 6 9 12 15 18 21 24 27
3 6 9 12 15 18 21
His1
2L
2R
3L
3R
X
3 6 9 12 15 18 21
2 4 6 8 10 12 14 16 18 20
3 6 9 12 15 18 21
3 6 9 12 15 18 21 24 27
3 6 9 12 15 18 21
HP1 (BPYE)
2L
2R
3L
3R
X
3 6 9 12 15 18 21
2 4 6 8 10 12 14 16 18 20
3 6 9 12 15 18 21
3 6 9 12 15 18 21 24 27
3 6 9 12 15 18 21

Figure S1

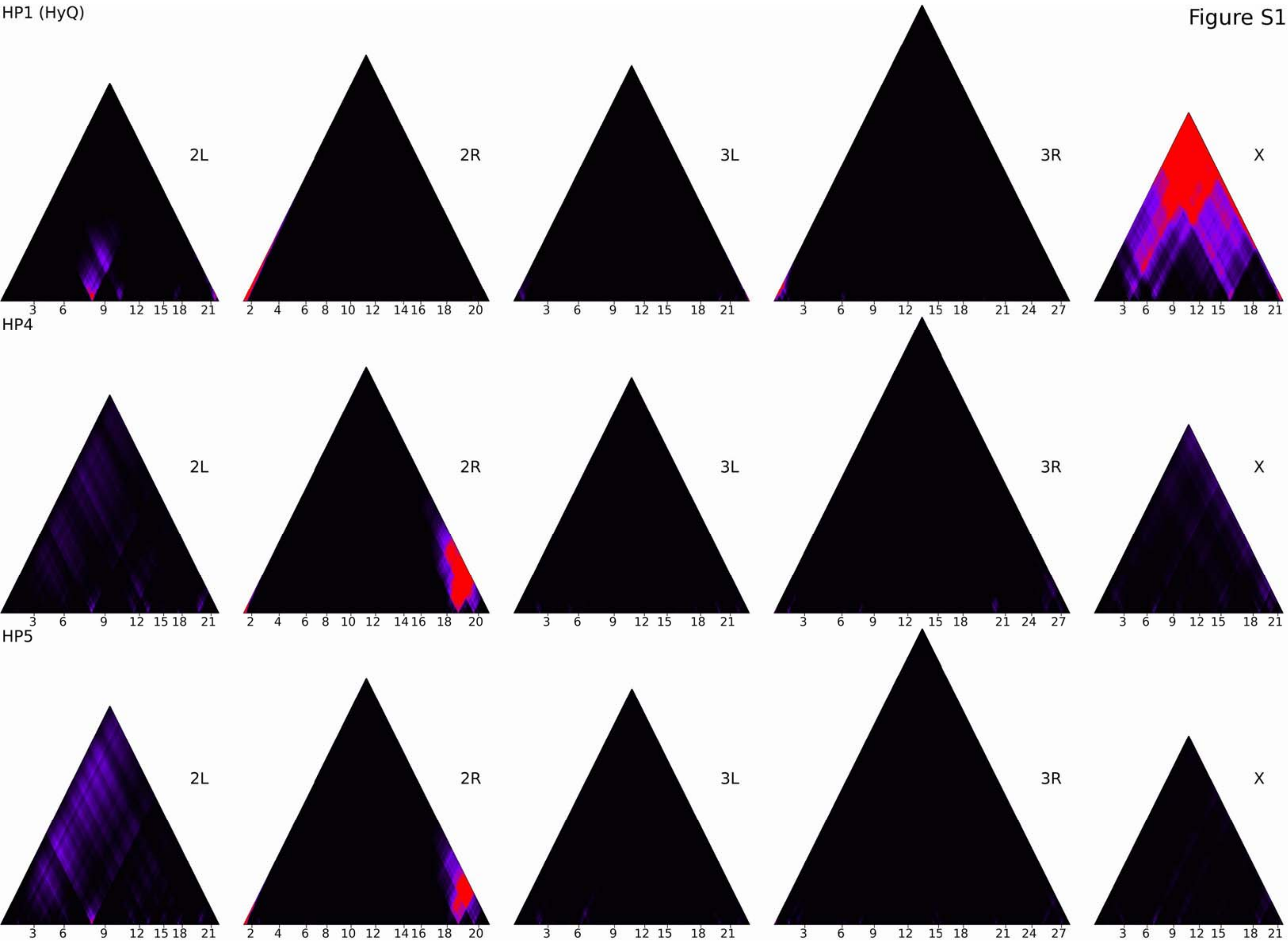
HP1 (HyQ)
HP4
HP5
2L
2R
3L
3R
X
3 6 9 12 15 18 21
2 4 6 8 10 12 14 16 18 20
3 6 9 12 15 18 21
3 6 9 12 15 18 21 24 27
3 6 9 12 15 18 21

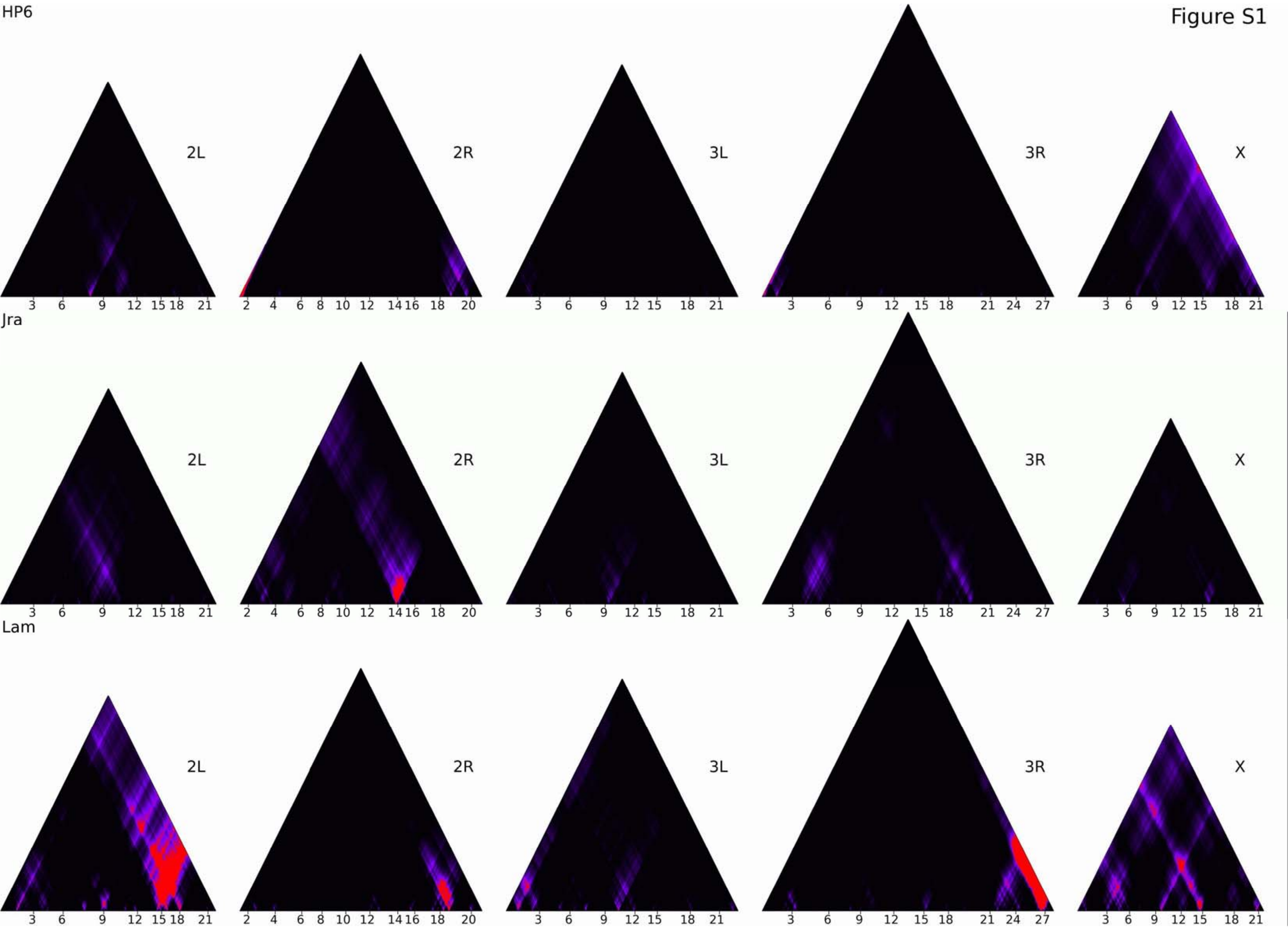
HP6
Jra
Lam
2L
2R
3L
3R
X
3 6 9 12 15 18 21
2 4 6 8 10 12 14 16 18 20
3 6 9 12 15 18 21
3 6 9 12 15 18 21 24 27
3 6 9 12 15 18 21

Figure S1

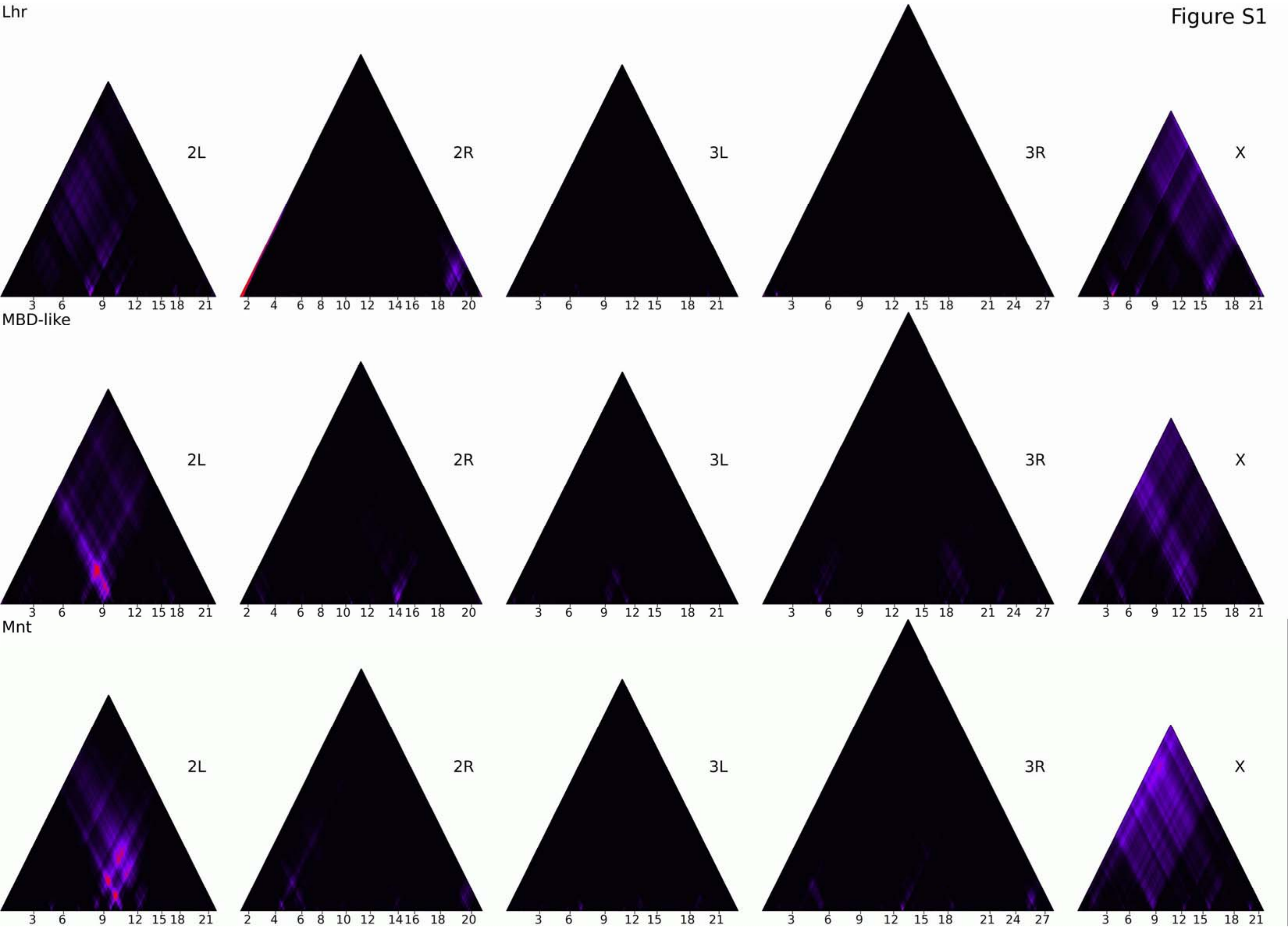
Figure S1
Lhr
2L
2R
3L
3R
X
MBD-like
Mnt
3 6 9 12 15 18 21
2 4 6 8 10 12 14 16 18 20
3 6 9 12 15 18 21
3 6 9 12 15 18 21 24 27
3 6 9 12 15 18 21

Figure S1

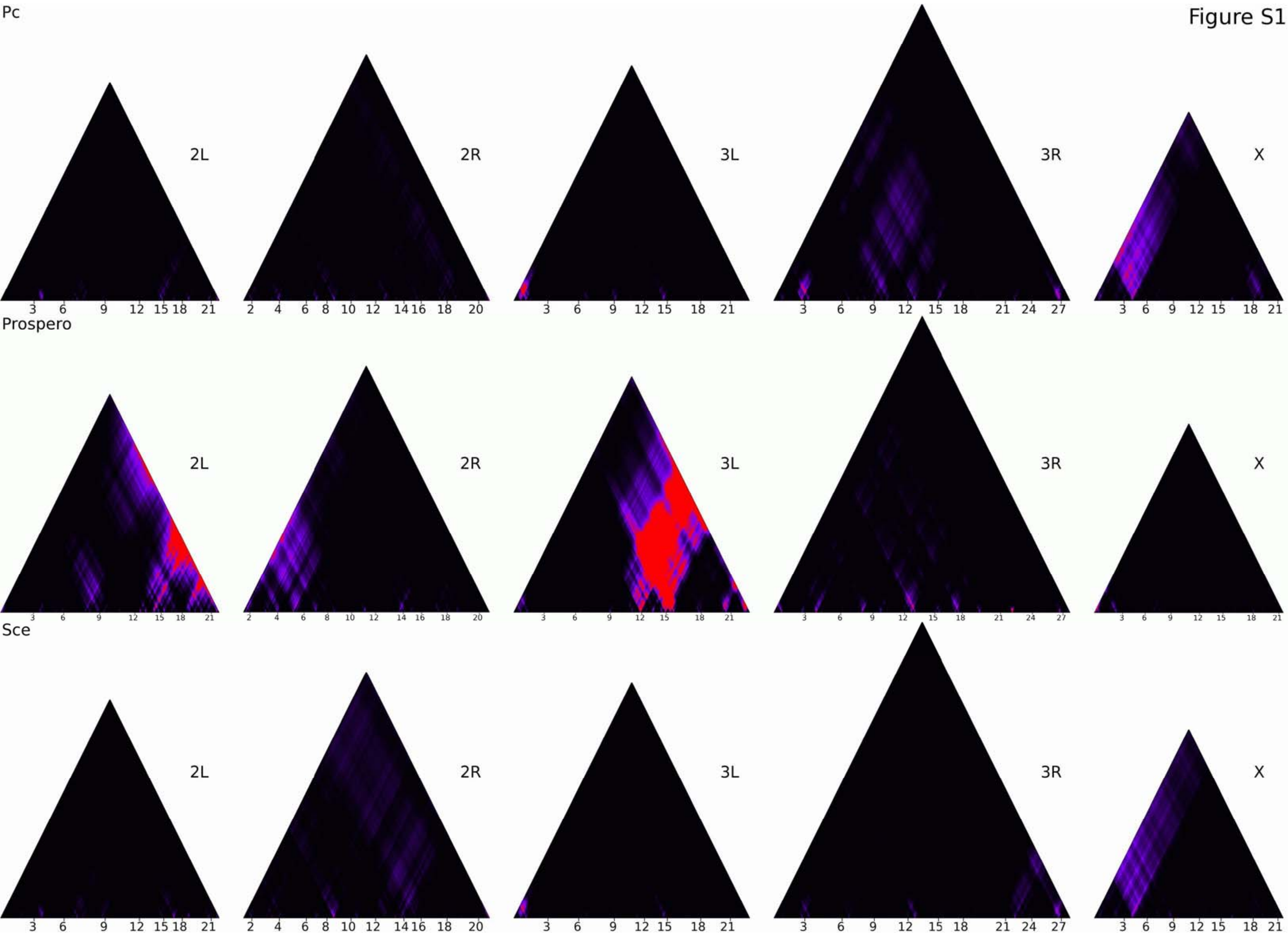

Figure S1

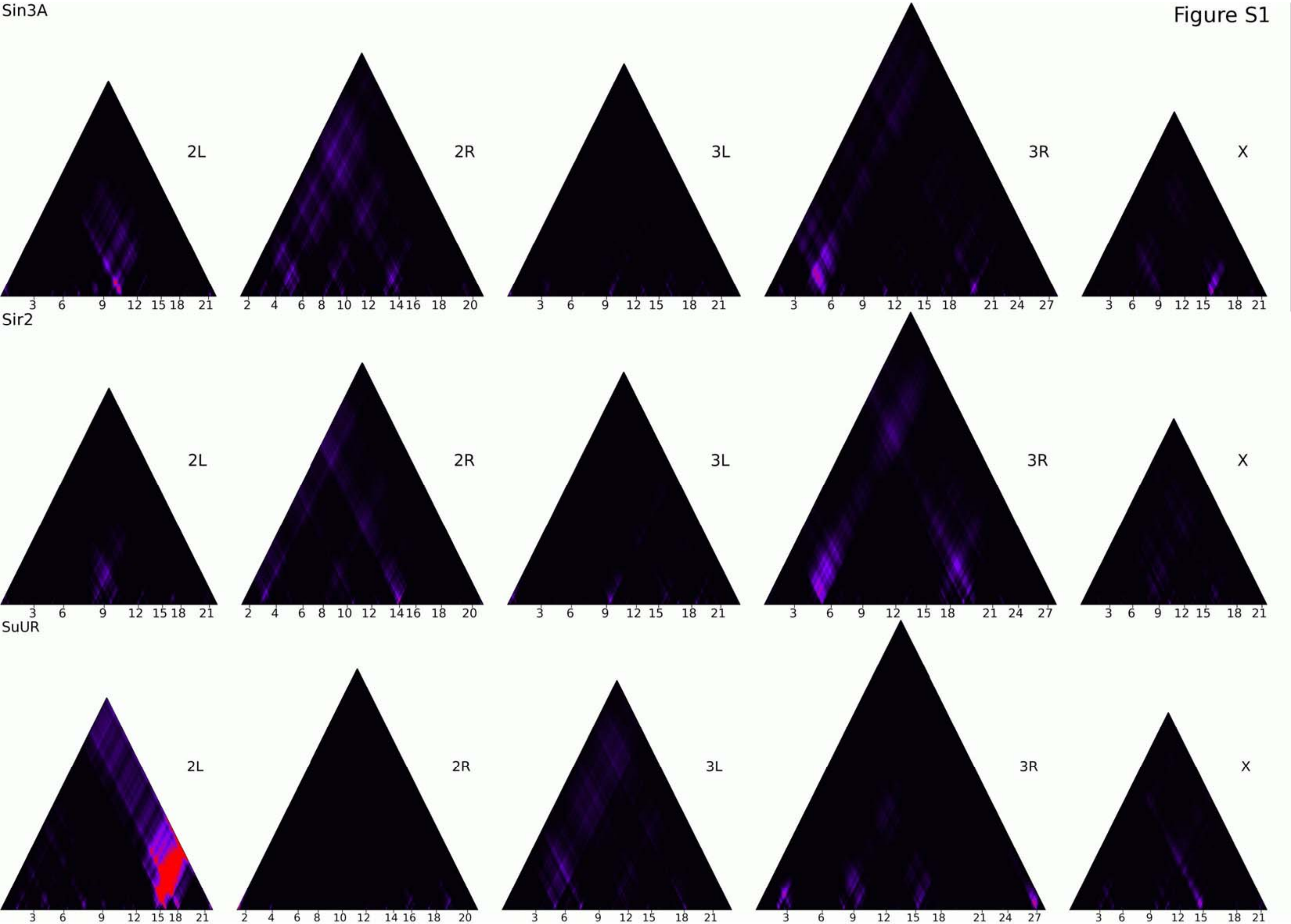
Sin3A
2L
2R
3L
3R
X
3 6 9 12 15 18 21
2 4 6 8 10 12 14 16 18 20
3 6 9 12 15 18 21
3 6 9 12 15 18 21 24 27
3 6 9 12 15 18 21
Sir2
2L
2R
3L
3R
X
3 6 9 12 15 18 21
2 4 6 8 10 12 14 16 18 20
3 6 9 12 15 18 21
3 6 9 12 15 18 21 24 27
3 6 9 12 15 18 21
SuUR
2L
2R
3L
3R
X
3 6 9 12 15 18 21
2 4 6 8 10 12 14 16 18 20
3 6 9 12 15 18 21
3 6 9 12 15 18 21 24 27
3 6 9 12 15 18 21

Figure S1

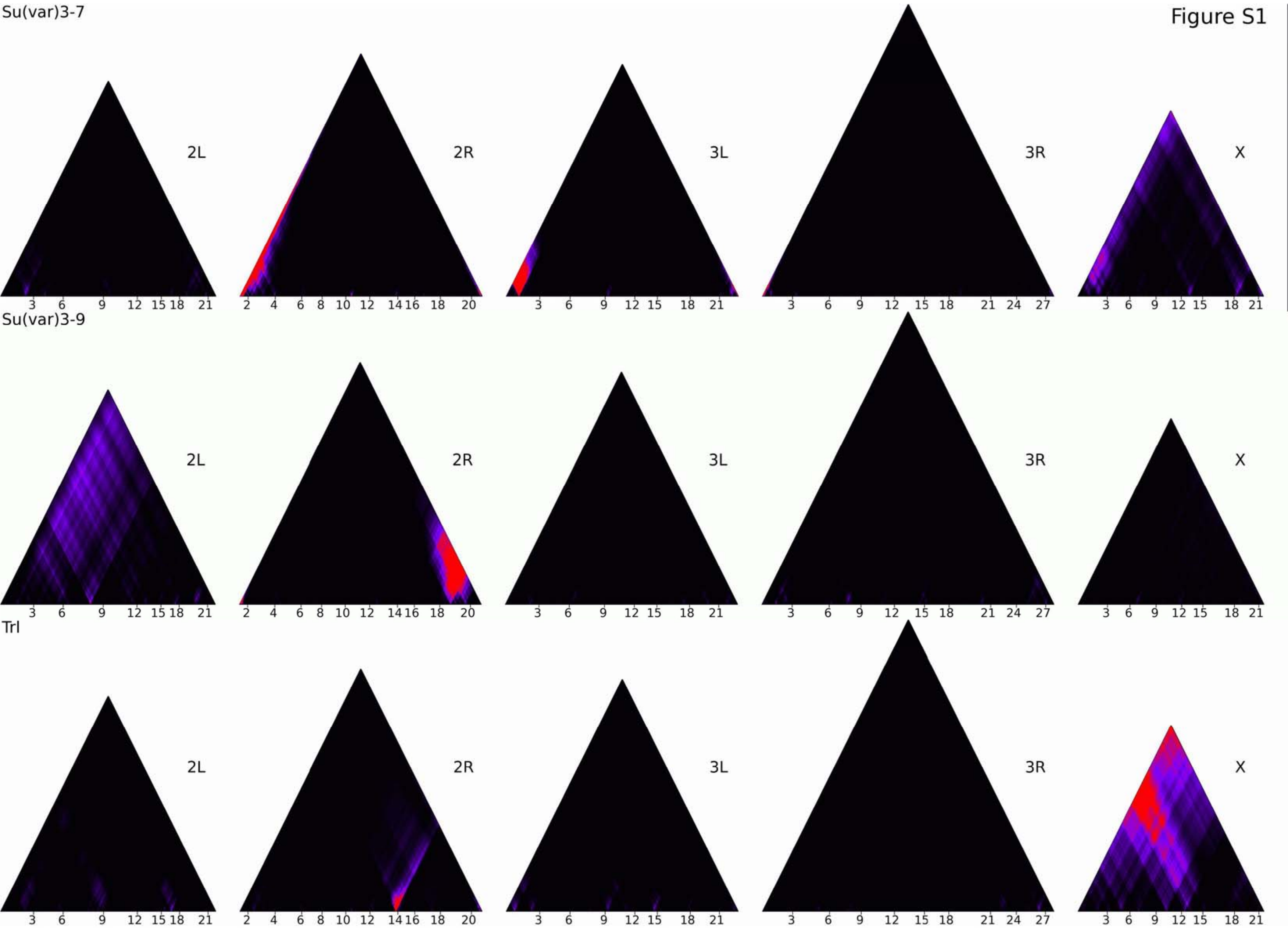
Su(var)3-7
2L
2R
3L
3R
X
Su(var)3-9
2L
2R
3L
3R
X
Trl
2L
2R
3L
3R
X
3 6 9 12 15 18 21
2 4 6 8 10 12 14 16 18 20
3 6 9 12 15 18 21
3 6 9 12 15 18 21 24 27
3 6 9 12 15 18 21
