# Supplementary figures and images for: Global Chromatin Domain Organization of the Drosophila Genome
Source: PLoS Genet. 2008 Mar 28;4(3):e1000045. doi: 10.1371/journal.pgen.1000045 (PMC2274884; doi:10.1371/journal.pgen.1000045)

Figure S2

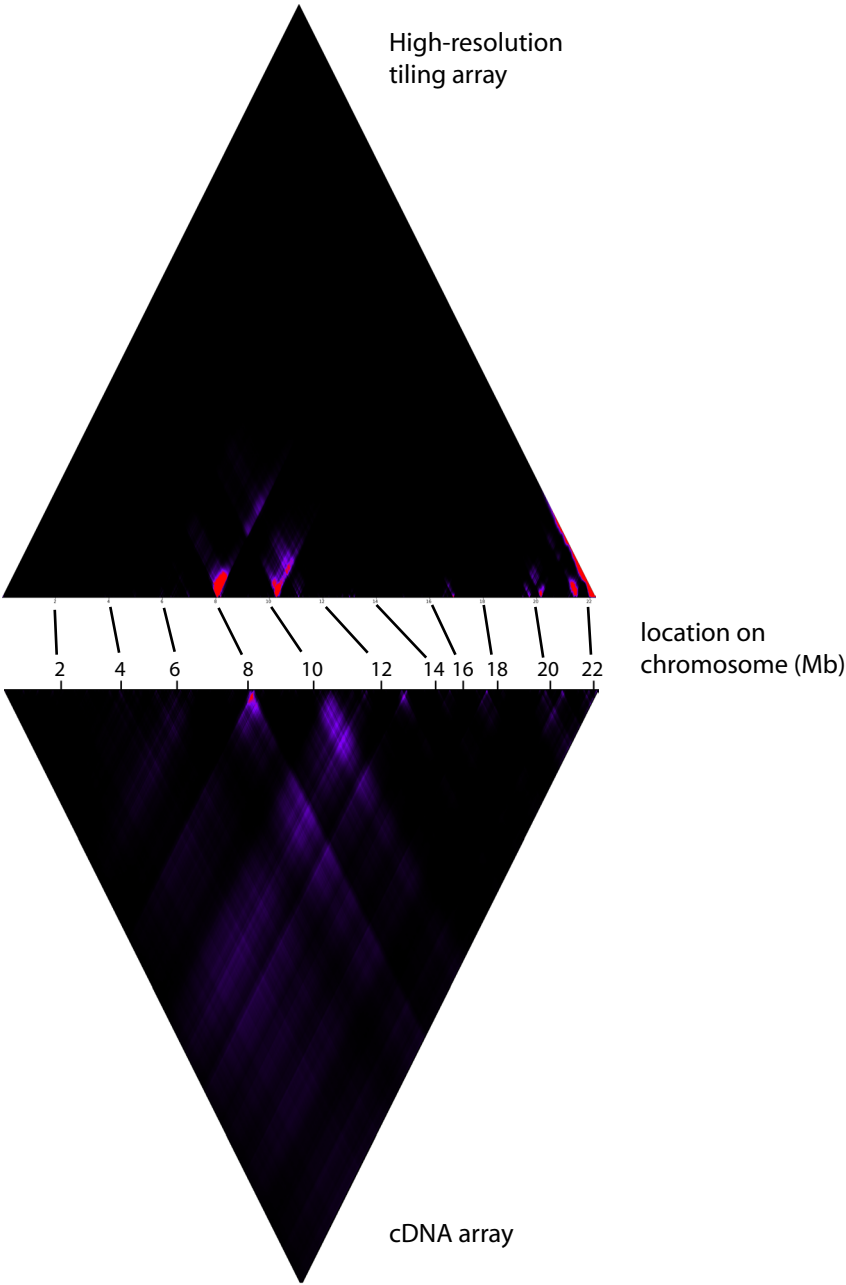

Supplement: Figure S2 — Comparison of cDNA data with high-resolution tiling array data. Domainograms for high resolution tiling array DamID data (top) and cDNA array DamID data (bottom) for HP1 on chromosome 2L. (0.81 MB PDF) [file pgen.1000045.s002.pdf]

Figure S3

A

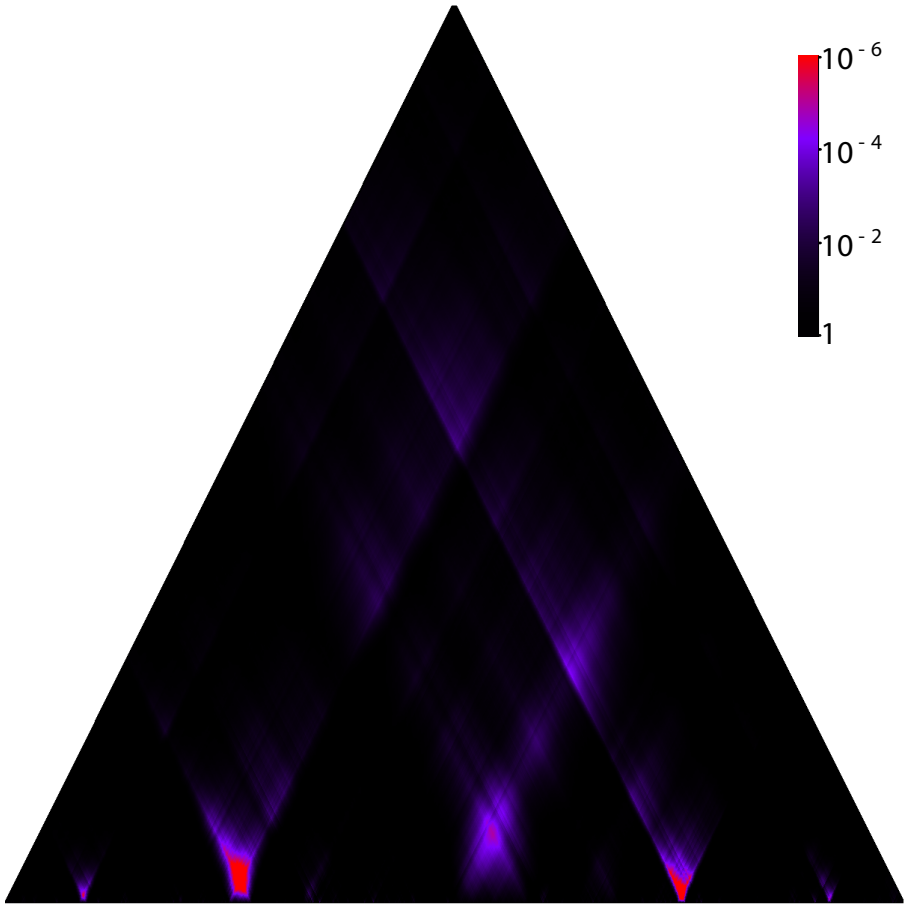

B

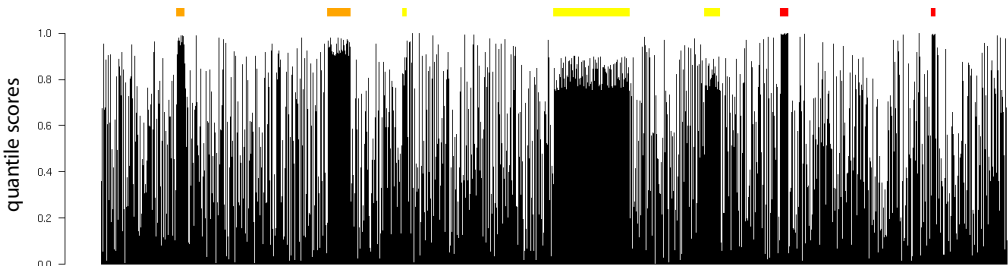

C

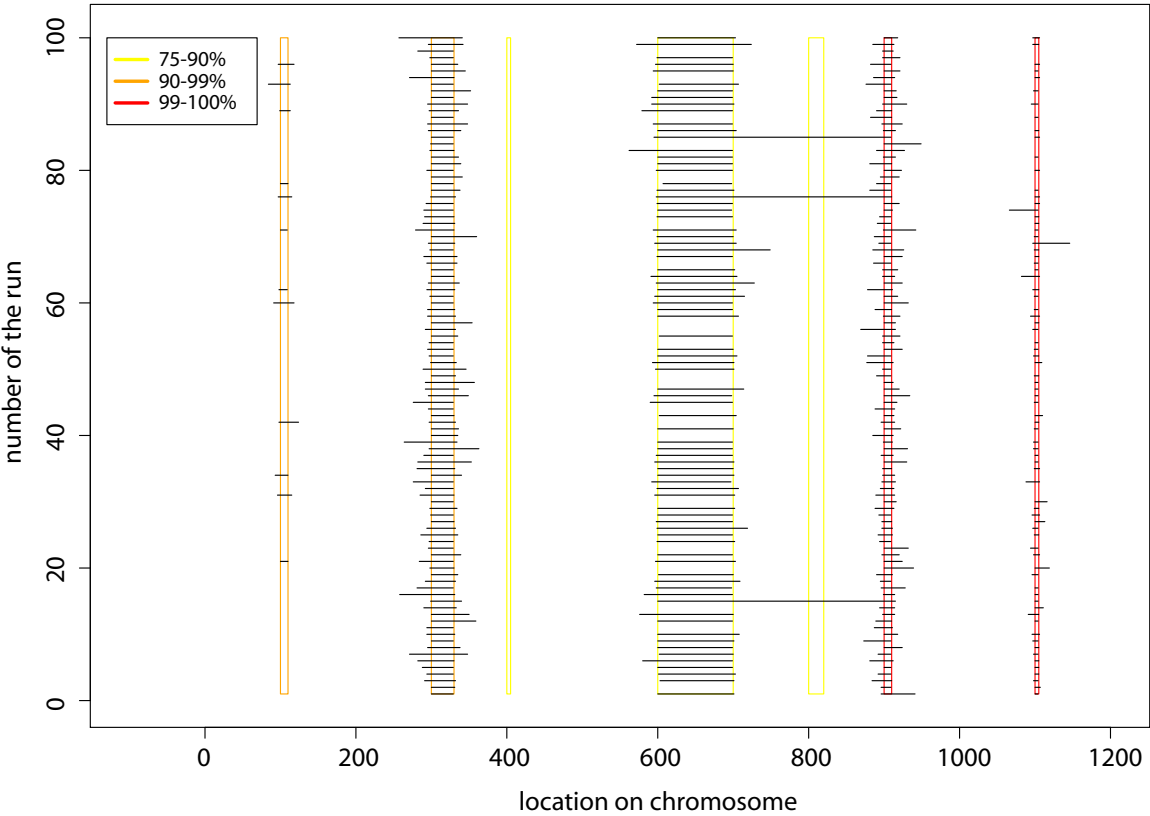

Supplement: Figure S3 — Domainogram and BRICK identification from synthetic data. Simulated data were generated to test the visualization and detection of chromatin domains. We created a virtual chromosome arm of 1200 genes, each associated with a quantile score (range 0–1) representing the ranked binding of a virtual protein. On this chromosome arm we placed seven domains consisting of 5–100 neighboring genes that were assigned quantile scores representing either “strong” (randomly selected quantile scores 0.99–1.00), “medium” (0.90–0.99) or “weak” (0.75–0.90) binding. The remainder of the genes was assigned a random value. A) Domainogram derived from an artificial dataset, and B) the corresponding simulated data. Yellow, orange and red rectangles denote the domains of weak, medium, and strong binding, respectively. C) Plot showing the performance of BRICK detection on 100 separate simulation runs. Horizontal lines denote the coordinates of identified BRICKs in each simulation run (vertical axis). Sensitivity of BRICK detection depends on the size and intensity of the domain, but identification of spurious domains or fusion of separate domains occurs very rarely. (0.75 MB PDF) [file pgen.1000045.s003.pdf]

de Wit et al, Supplementary Figure S5

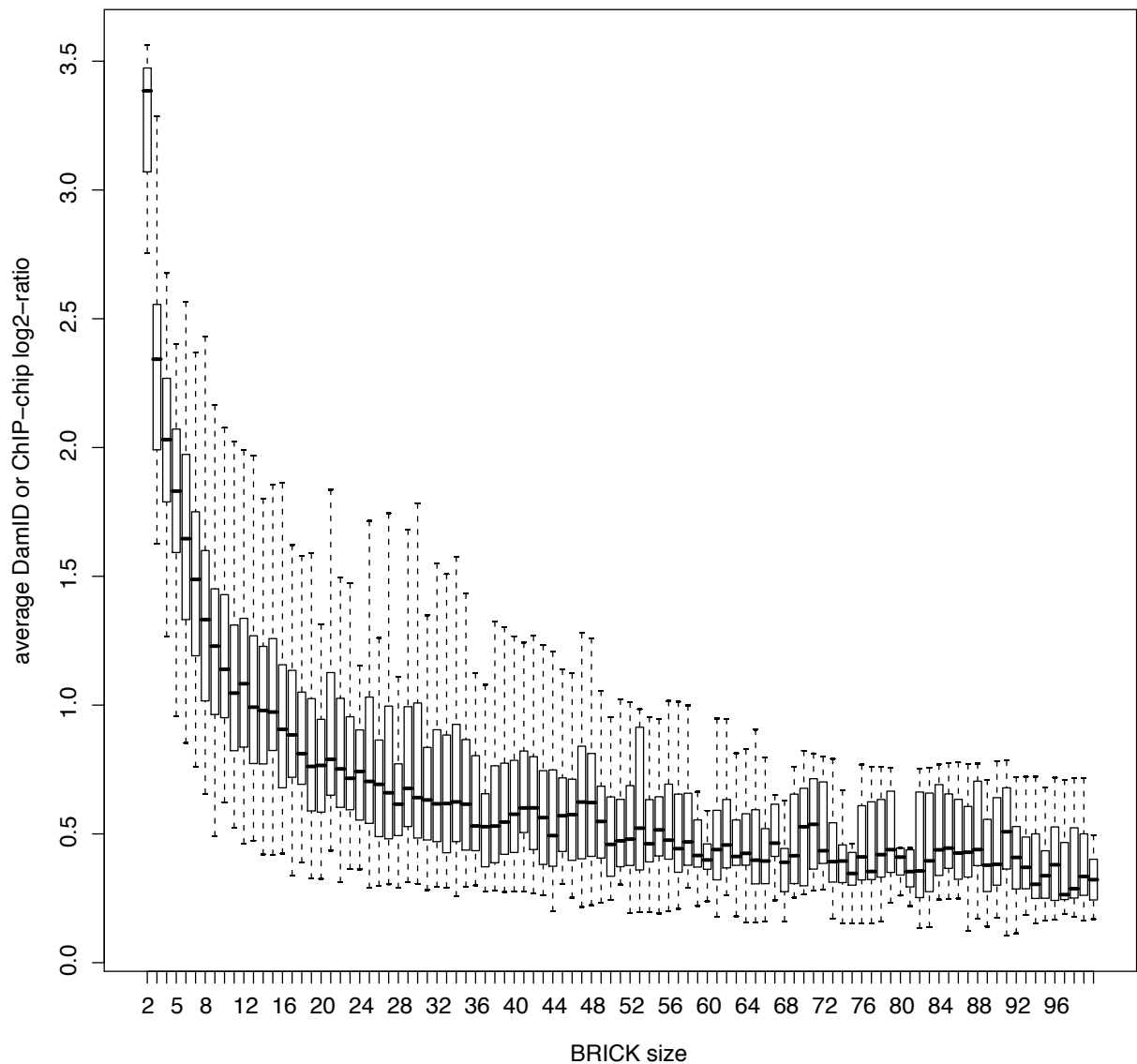

Supplement: Figure S5 — Enrichments of protein binding in BRICKs. Boxplots are shown of the average protein binding (DamID or ChIP) logratio for each BRICK size. Boxes show 25th–75th percentile, and the horizontal line inside each box indicates the median. (0.16 MB PDF) [file pgen.1000045.s005.pdf]

Figure S6

Bicoid

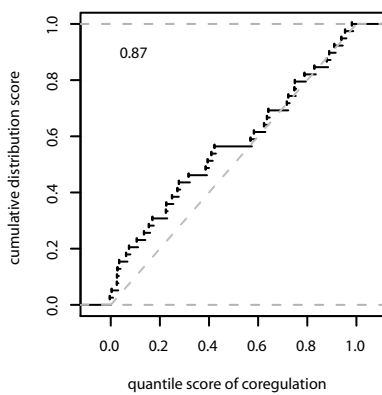

brahma

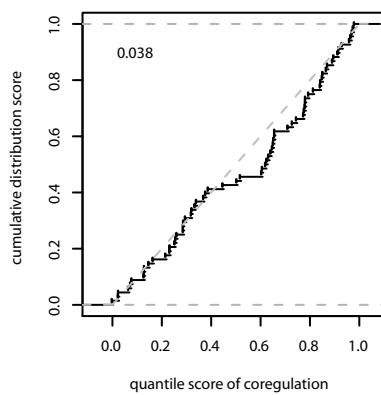

D1

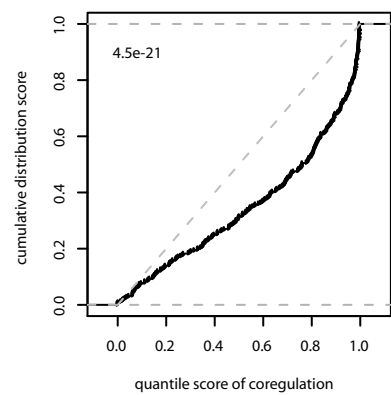

DSP1

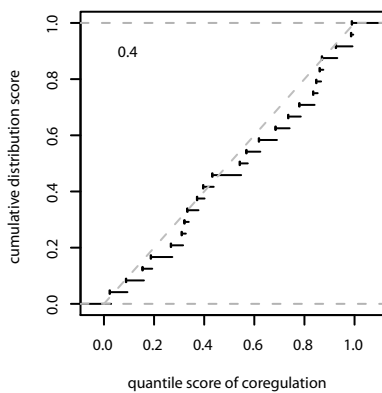

esc

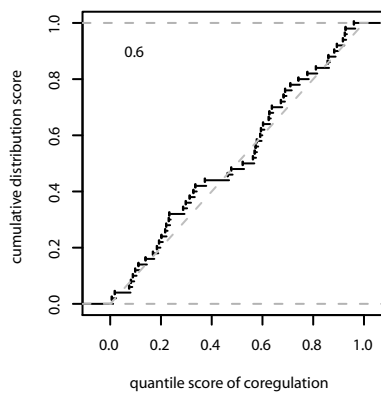

Eve

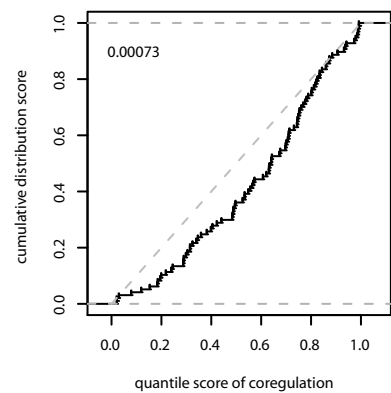

Groucho

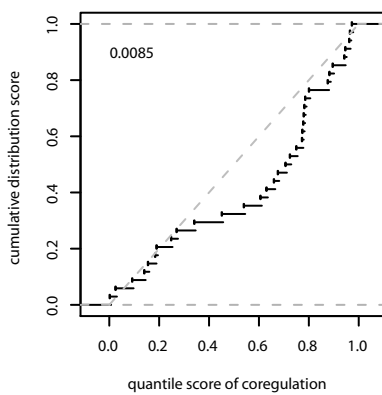

H1

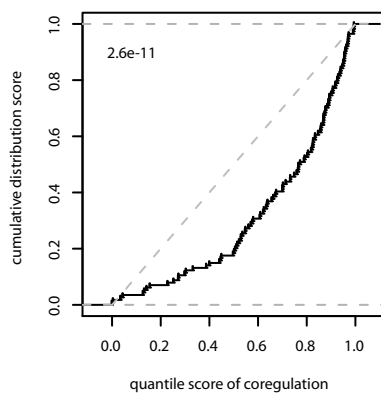

H3.3

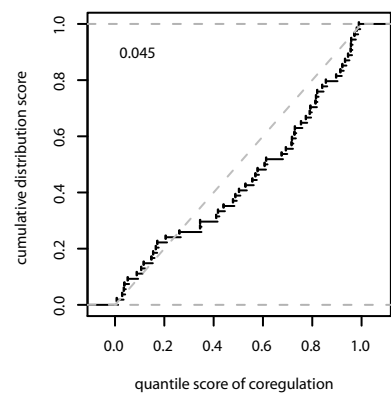

H3K27me3

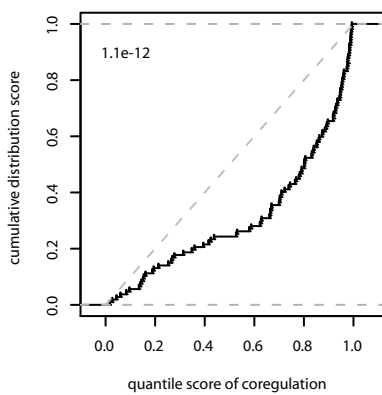

H3K4me3

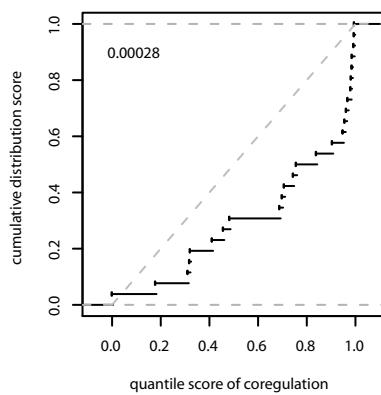

HP1 (BPYE)

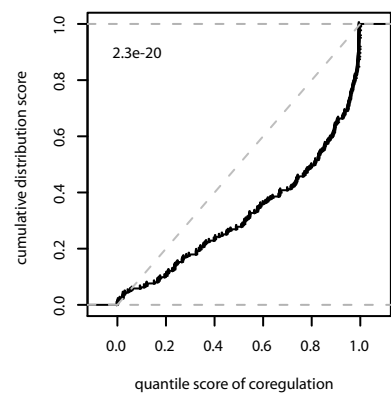

HP1 (HyQ)

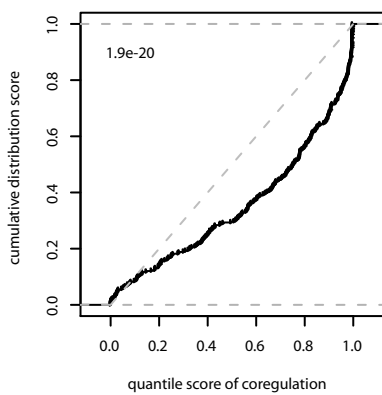

HP4

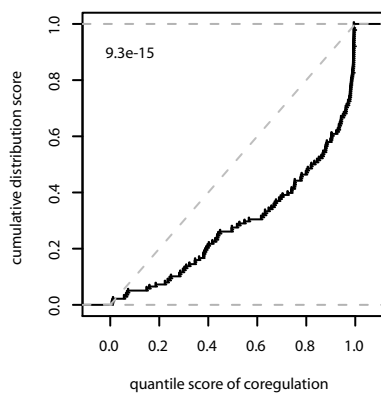

HP5

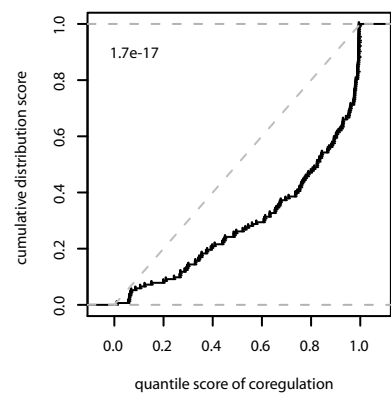

HP6

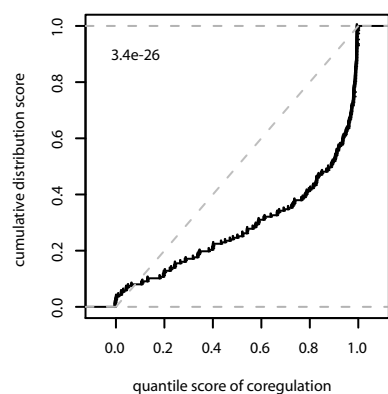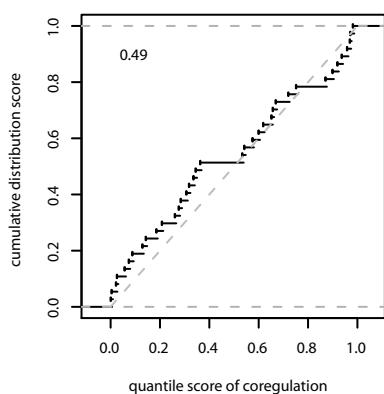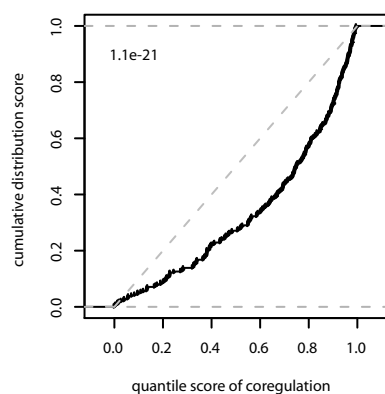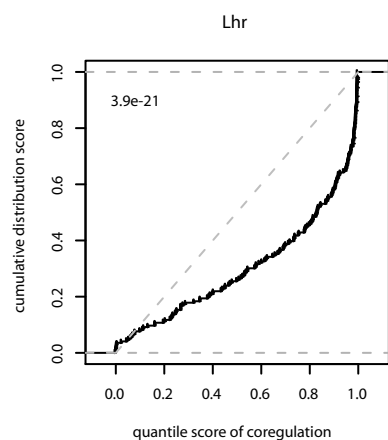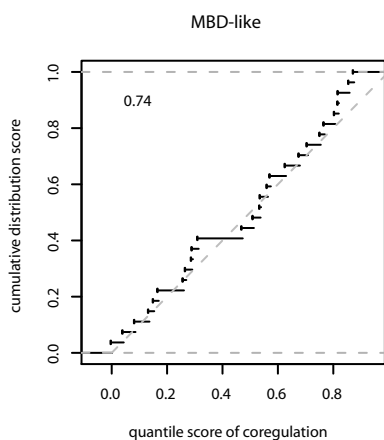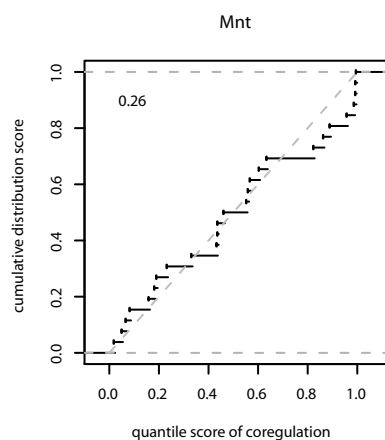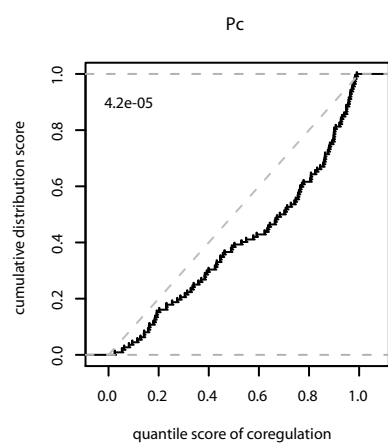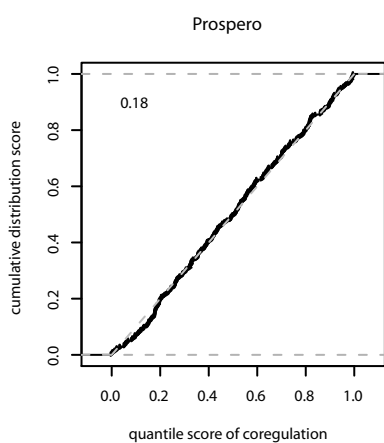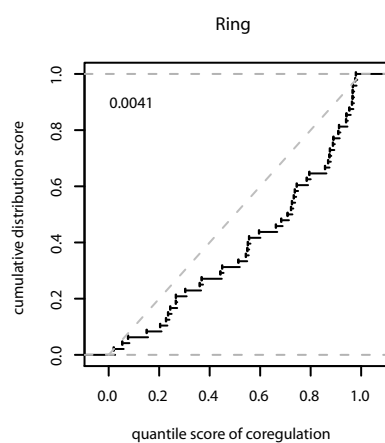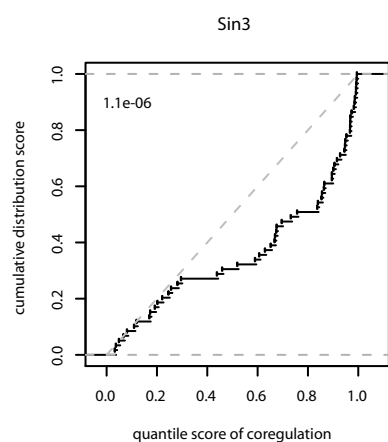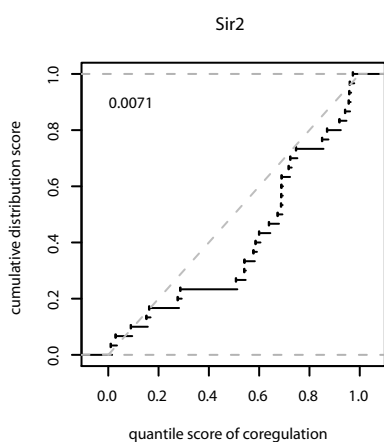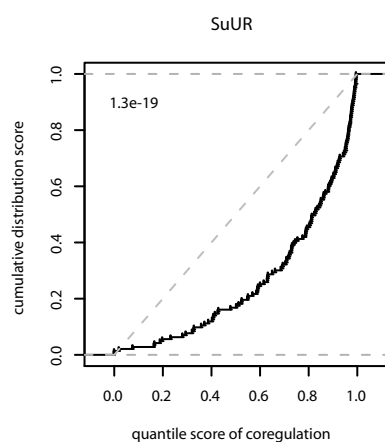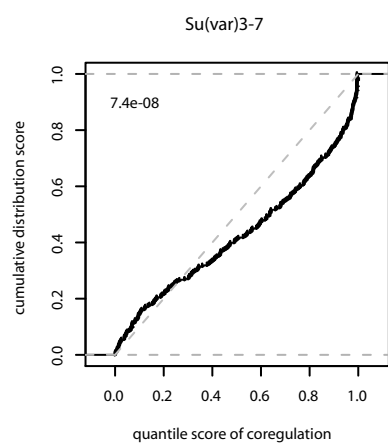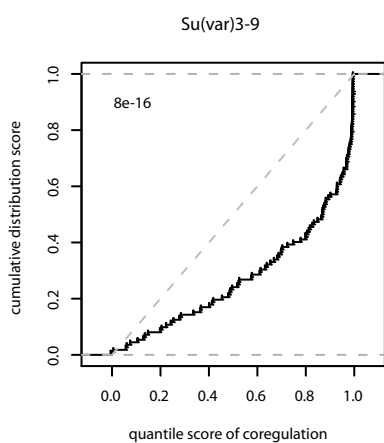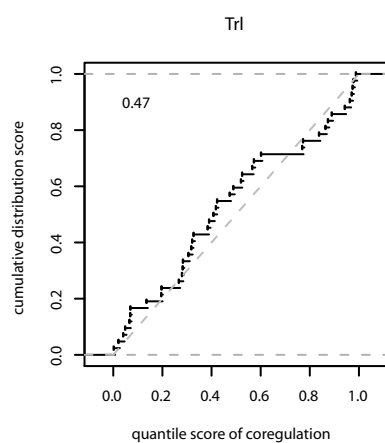

Supplement: Figure S6 — Empirical cumulative distribution plots for quantile scores for coregulation in domains. Figures show cumulative distribution of quantile scores of coregulation (see Text S1 for details on the calculation of quantile scores). Each figure represents the coregulation level for one protein as indicated. Horizontal axes represent quantile scores, vertical axes represent the relative cumulative level for a given quantile score. The dashed gray line represents the theoretical uniform distribution. In the top-left corner of each graph is indicated the p-value according to the KS-test, for deviation from a uniform distribution. (0.94 MB PDF) [file pgen.1000045.s006.pdf]

Supplementary Figure S7

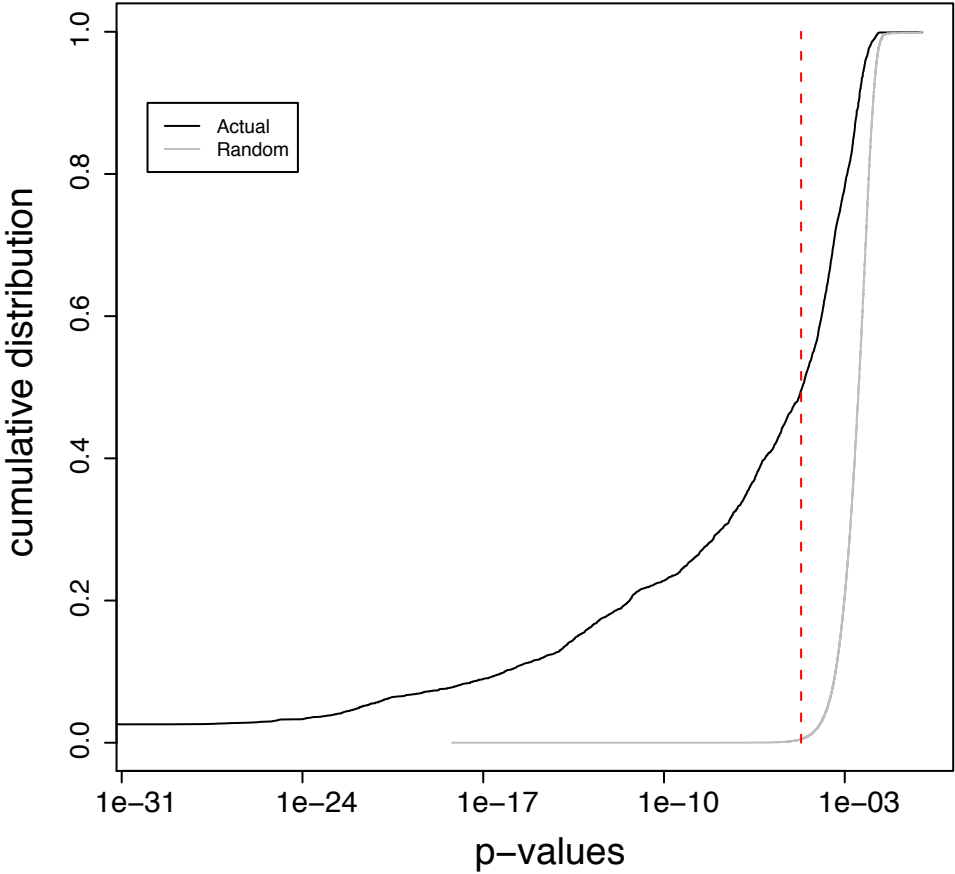

Supplement: Figure S7 — Empirical cumulative distribution of p-values for enrichment of GO categories. P-values of enrichment for GO categories were calculated using the cumulative hypergeometric distribution. Empirical distribution of the p-values in the domains is shown in black. The gray line denotes the empirical distribution of p-values from 10,000 randomized genomes. The red dashed line denotes the p-values for which the FDR is 0.01. (0.27 MB PDF) [file pgen.1000045.s007.pdf]

Figure S8 A

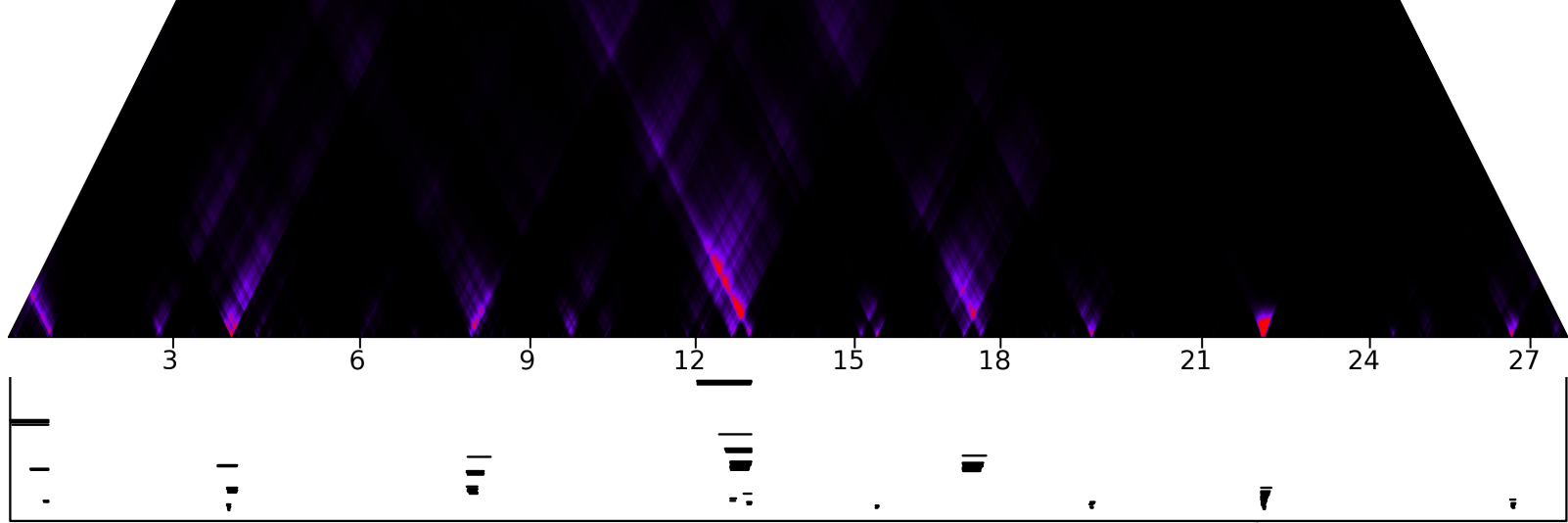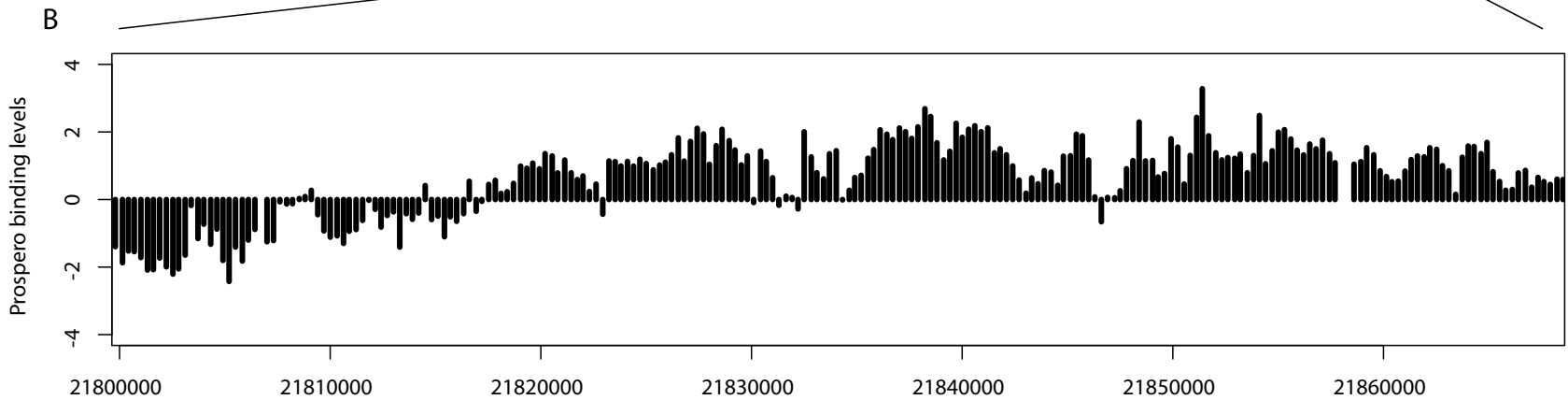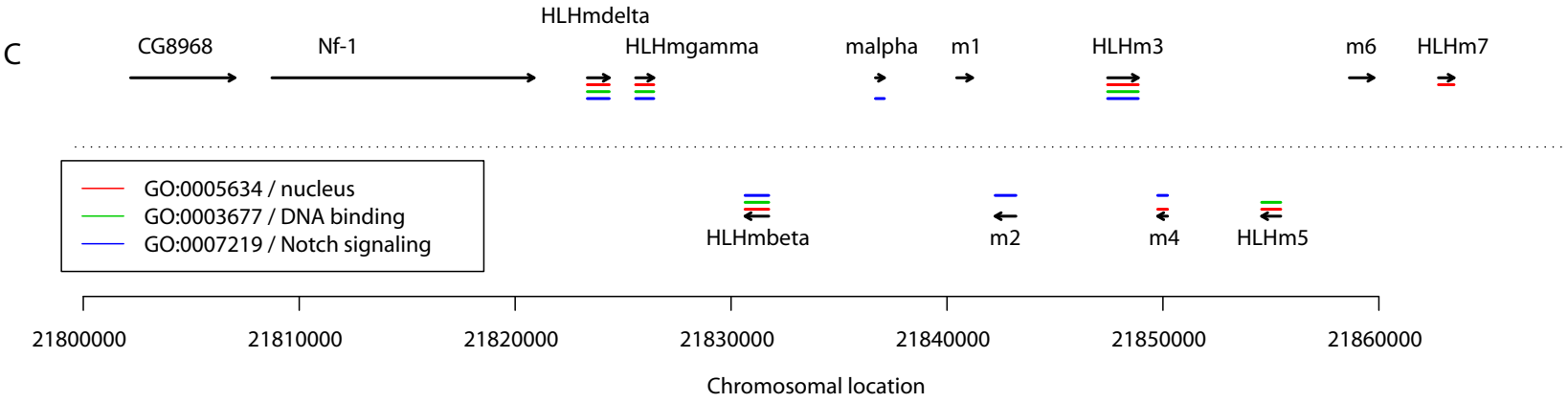

Supplement: Figure S8 — A Prospero chromatin domain is enriched for genes encoding transcription factors involved in Notch signaling. A) Bottom part of a domainogram of chromosome 3R for Prospero binding. Below the plot the corresponding BRICK structure is shown. B) Chromosomal map showing tiling array data with log2 binding ratios for Prospero (Choksi et al. Dev Cell. 2006 Dec;11(6):775-89) in a BRICK region. C) Genes located in the same region. Three major GO categories are indicated by different colors. (1.07 MB PDF) [file pgen.1000045.s008.pdf]

Figure S9

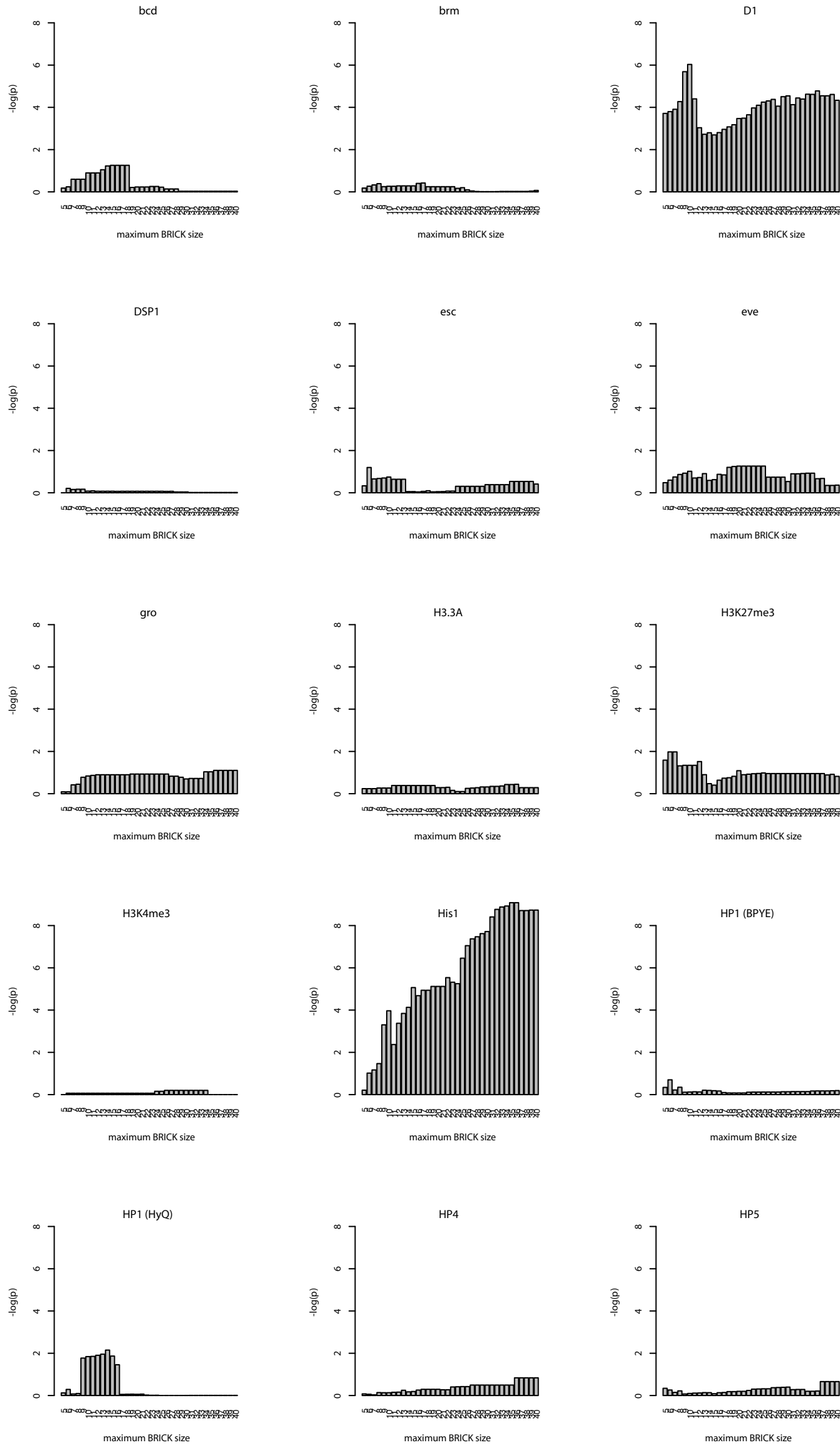

Figure S8

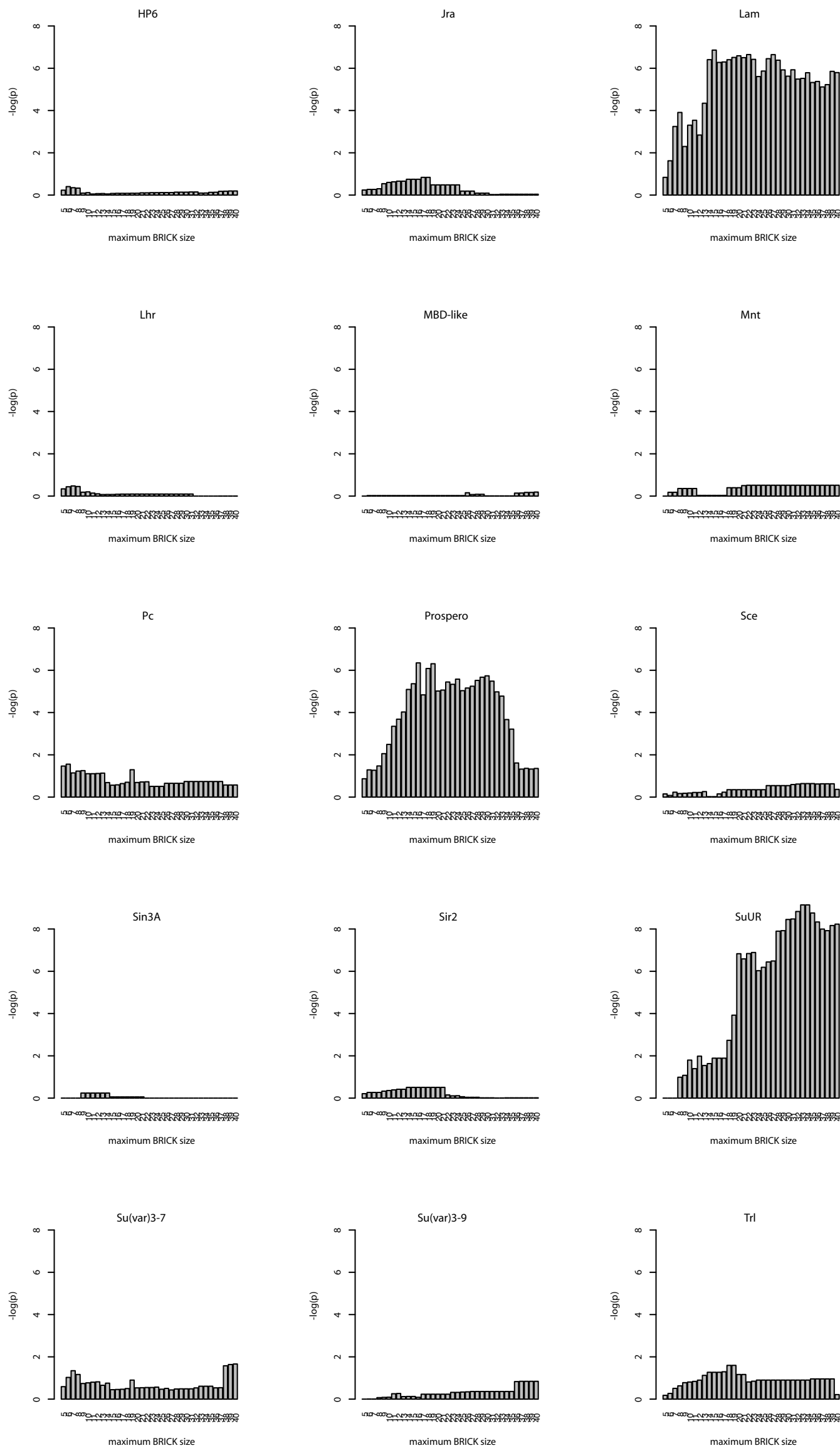

Supplement: Figure S9 — Synteny breakpoints are significantly depleted from BRICKs defined by some proteins. Depletion of synteny breakpoints from BRICKs is calculated using the cumulative hypergeometric distribution. For every protein, barplots show the p-value as a function of the maximum BRICK size. For a maximum BRICK size, all BRICKs up to that size are included. (0.47 MB PDF) [file pgen.1000045.s009.pdf]
