# Supplementary material for: Global Chromatin Domain Organization of the Drosophila Genome
Source: PLoS Genet. 2008 Mar 28;4(3):e1000045. doi: 10.1371/journal.pgen.1000045 (PMC2274884; doi:10.1371/journal.pgen.1000045)
Supplement: Figure S4 — BRICK plots showing the distribution of chromatin domains for all proteins on each chromosome arm. BRICKs <100 probed genes are shown. Each horizontal line depicts the position of a BRICK. For each protein, the relative vertical location of the lines represents the number of probed genes in a BRICK. (0.38 MB PDF) [file pgen.1000045.s004.pdf]

Figure S4

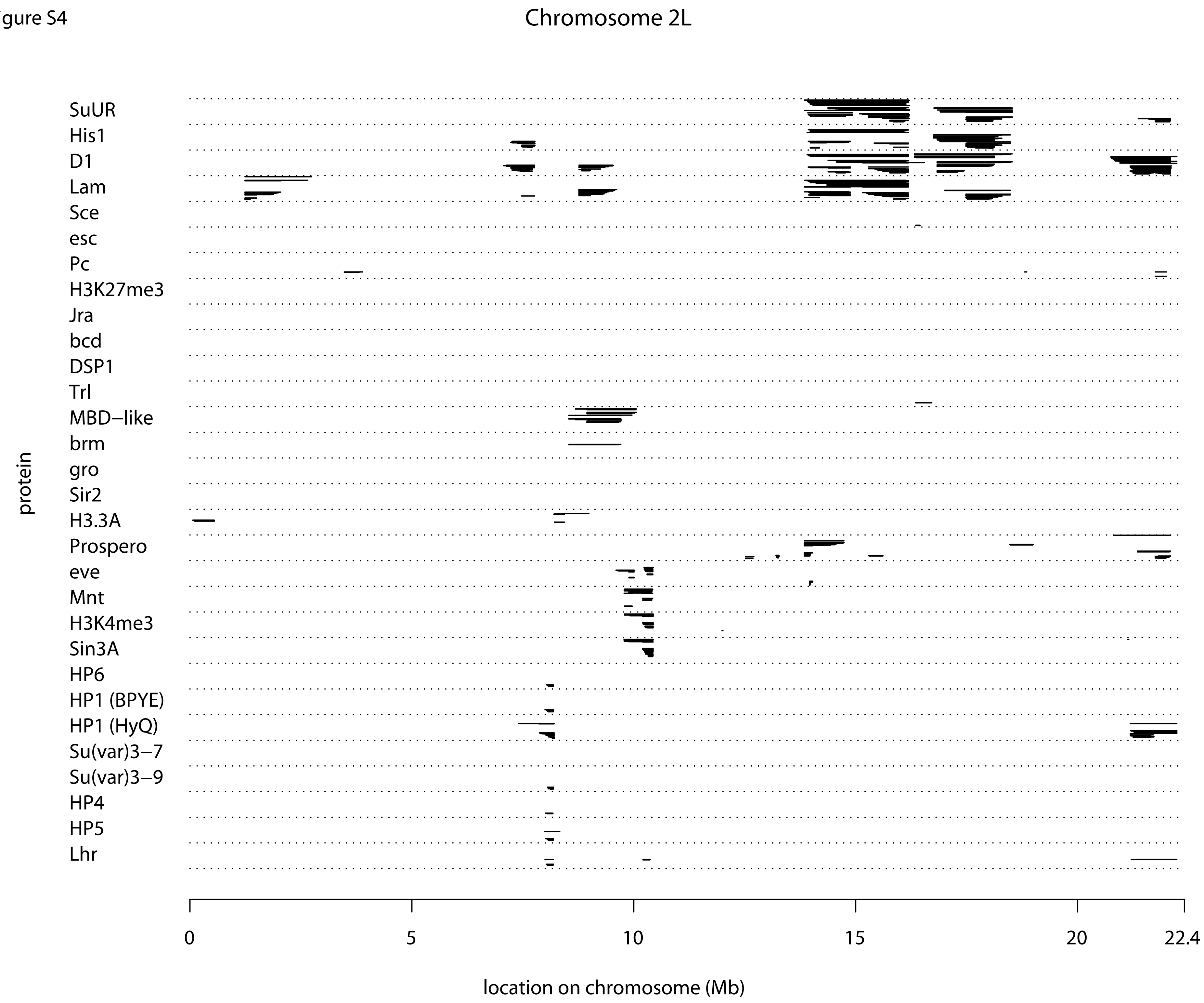
Chromosome 2L
SuUR
His1
D1
Lam
Sce
esc
Pc
H3K27me3
Jra
bcd
DSP1
Trl
MBD−like
brm
gro
Sir2
H3.3A
Prospero
eve
Mnt
H3K4me3
Sin3A
HP6
HP1 (BPYE)
HP1 (HyQ)
Su(var)3−7
Su(var)3−9
HP4
HP5
Lhr
protein
0
5
10
15
20
22.4
location on chromosome (Mb)

Figure S4

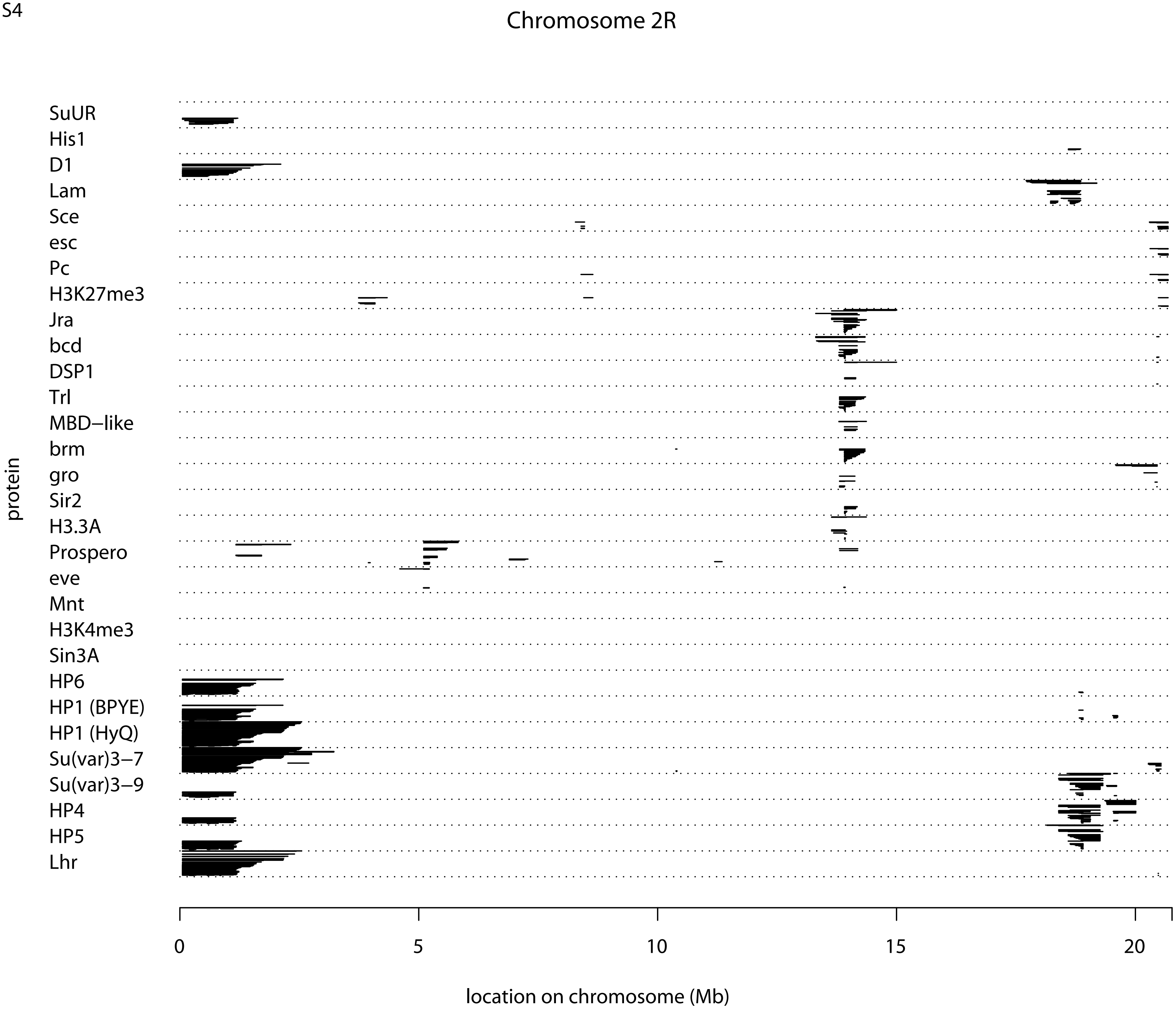

Chromosome 2R
SuUR
His1
D1
Lam
Sce
esc
Pc
H3K27me3
Jra
bcd
DSP1
Trl
MBD−like
brm
gro
Sir2
H3.3A
Prospero
eve
Mnt
H3K4me3
Sin3A
HP6
HP1 (BPYE)
HP1 (HyQ)
Su(var)3−7
Su(var)3−9
HP4
HP5
Lhr
protein
0
5
10
15
20
location on chromosome (Mb)

Figure S4

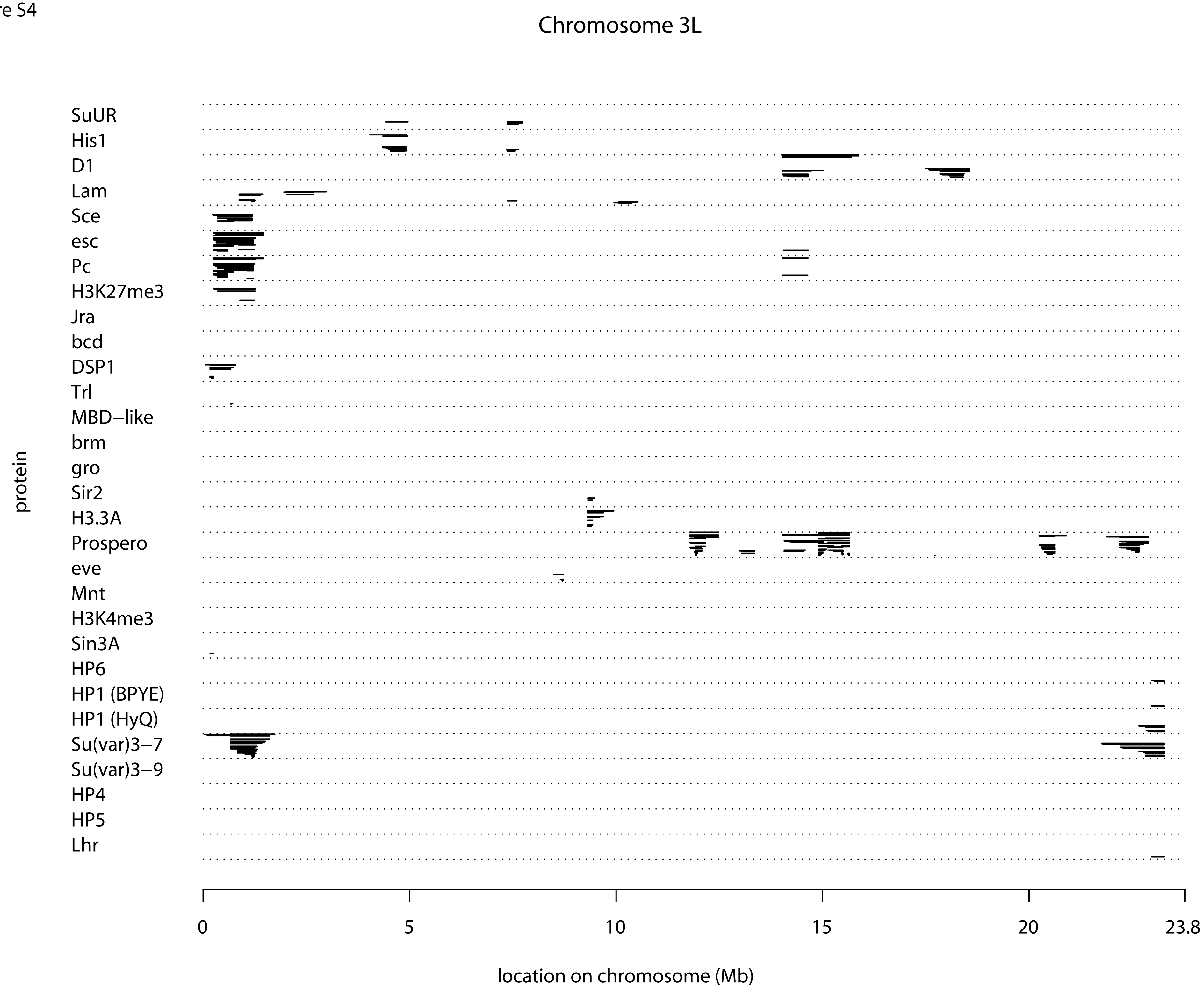
Chromosome 3L
SuUR
His1
D1
Lam
Sce
esc
Pc
H3K27me3
Jra
bcd
DSP1
Trl
MBD−like
brm
gro
Sir2
H3.3A
Prospero
eve
Mnt
H3K4me3
Sin3A
HP6
HP1 (BPYE)
HP1 (HyQ)
Su(var)3−7
Su(var)3−9
HP4
HP5
Lhr
protein
0
5
10
15
20
23.8
location on chromosome (Mb)

Figure S4

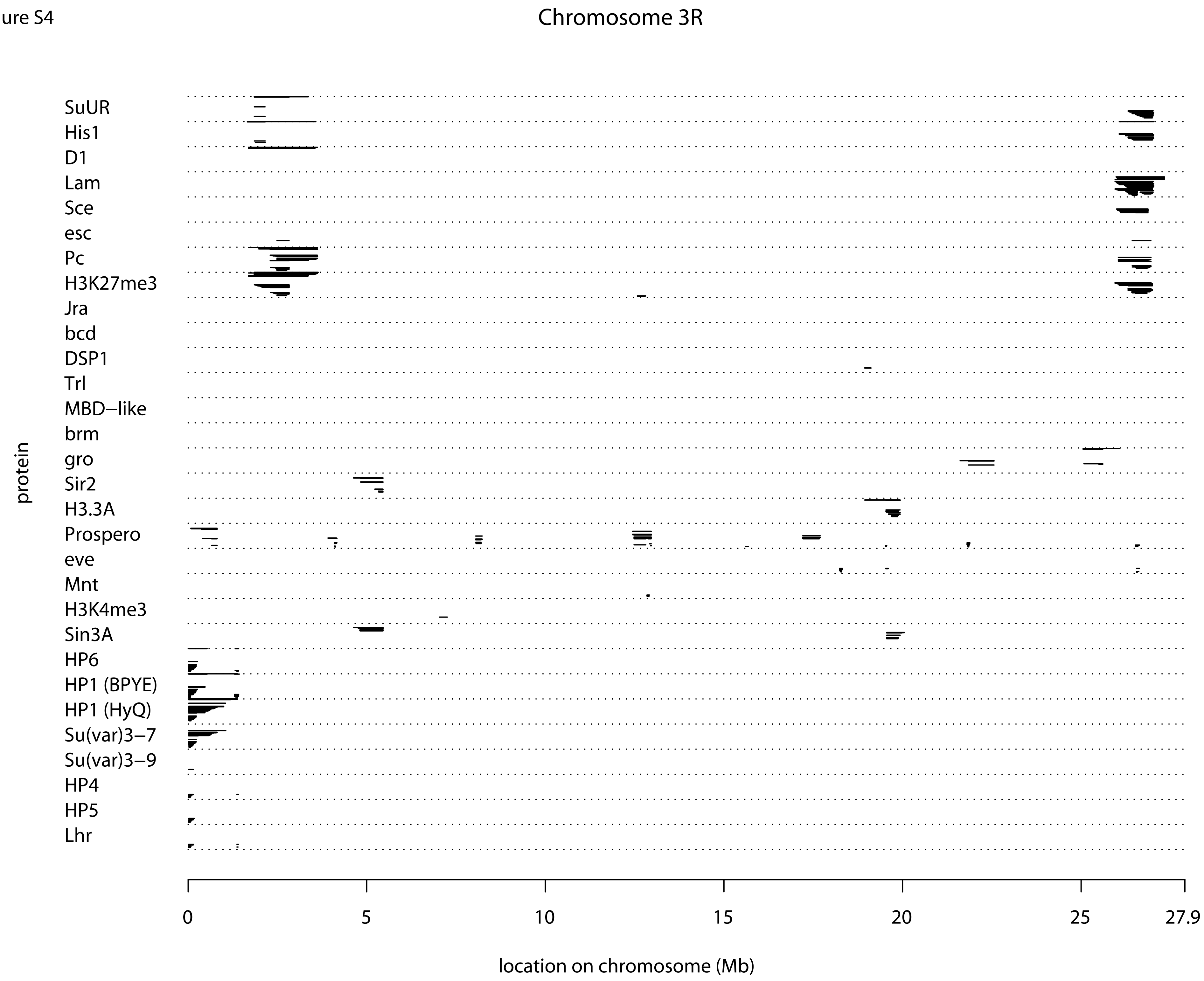
Chromosome 3R
SuUR
His1
D1
Lam
Sce
esc
Pc
H3K27me3
Jra
bcd
DSP1
Trl
MBD−like
brm
gro
Sir2
H3.3A
Prospero
eve
Mnt
H3K4me3
Sin3A
HP6
HP1 (BPYE)
HP1 (HyQ)
Su(var)3−7
Su(var)3−9
HP4
HP5
Lhr
protein
0
5
10
15
20
25
27.9
location on chromosome (Mb)

Figure S4

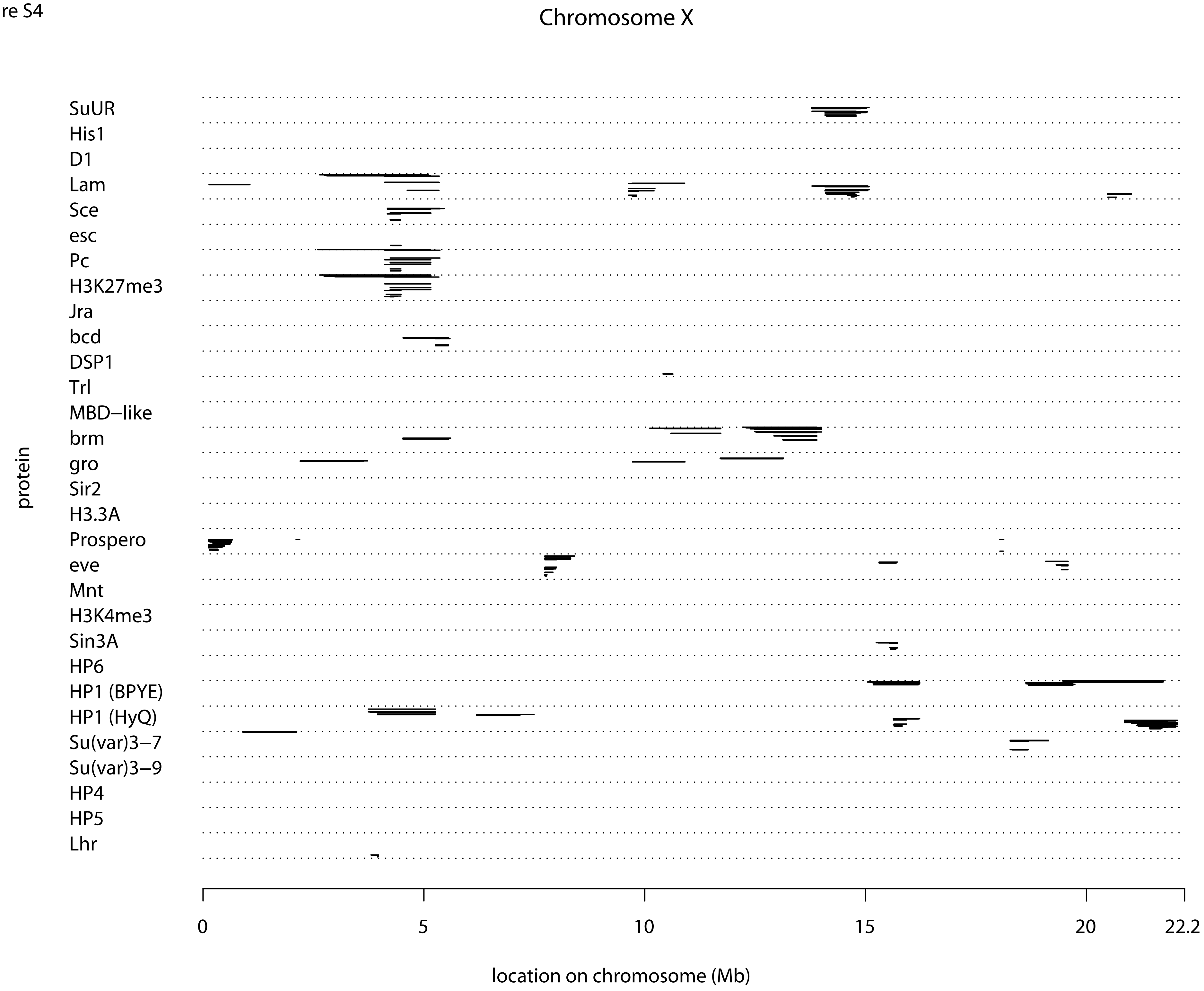

Chromosome X
SuUR
His1
D1
Lam
Sce
esc
Pc
H3K27me3
Jra
bcd
DSP1
Trl
MBD−like
brm
gro
Sir2
H3.3A
Prospero
eve
Mnt
H3K4me3
Sin3A
HP6
HP1 (BPYE)
HP1 (HyQ)
Su(var)3−7
Su(var)3−9
HP4
HP5
Lhr
protein
0
5
10
15
20
22.2
location on chromosome (Mb)

Figure S4

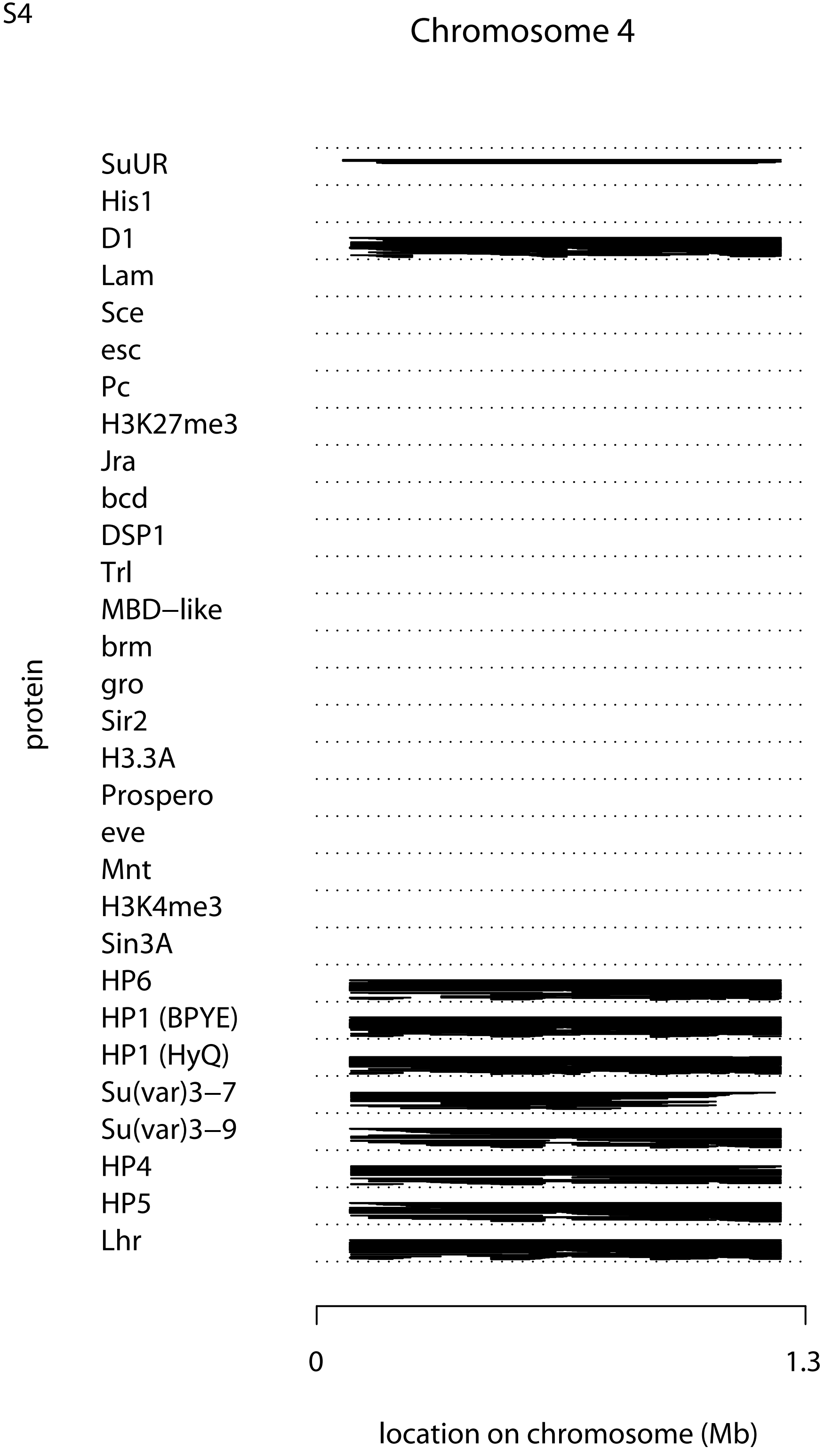

Chromosome 4
protein
SuUR
His1
D1
Lam
Sce
esc
Pc
H3K27me3
Jra
bcd
DSP1
Trl
MBD−like
brm
gro
Sir2
H3.3A
Prospero
eve
Mnt
H3K4me3
Sin3A
HP6
HP1 (BPYE)
HP1 (HyQ)
Su(var)3−7
Su(var)3−9
HP4
HP5
Lhr
0
1.3
location on chromosome (Mb)
