# Supplementary material for: Global Chromatin Domain Organization of the Drosophila Genome
Source: PLoS Genet. 2008 Mar 28;4(3):e1000045. doi: 10.1371/journal.pgen.1000045 (PMC2274884; doi:10.1371/journal.pgen.1000045)
Supplement: Text S1 — Additional information on computational methods. (0.57 MB PDF) [file pgen.1000045.s010.pdf]

Supplementary Methods

de Wit *et al*,

Global chromatin domain organization of the *Drosophila* genome

Pseudo-code describing the BRICK algorithm

```
#max is the number of probes on the chromosome
define an empty array for the probabilities of length max
define an empty array to hold the window lengths of length max
for i=1 until max
  w=1
  min_p, length_min_p #define the minimal pvalue and the length
                      #associated with it
  while(w <= i)
    calculate Qiw
    if w==1
      Qiw=Qiw**gamma
    Qiw=Qiw**w
    Qiw=Qiw*V_prob[w-i]
    if Qiw < min_p
      min_p, length_min_p=min_p, w
    w++
  V_length[i],V_prob[i]=length_min_p,min_p
```

Traceback:

```
w=max
while(w > 0)
  save domain length stored at V_length[w]
  decrement w by V_length[w]
```

Choosing the bias factor γ

We estimated the false discovery rate (FDR) of BRICK identification by determining the number of windows of size w > 1 identified in a random permutation of the genome. In a randomly permutated genome all domains identified are by definition false. Low values of γ favor parses consisting of *w*=1 windows. We seek an optimum such that the number of falsely

identified domains does not exceed 5% of the number of domains that we identify in an actual binding profile (*i.e.* FDR < 0.05).

To estimate the number of false positive BRICKs identified we calculated the optimal path through 1,000 randomly permutated genomes. We performed these analyses for various values of γ (in decreasing order: 0.01, 0.005, 0.001, 0.0005 and 0.0001) and scored the number of identified BRICKs, as well as the number of genes located within identified BRICKs. This was done for both the randomized datasets and the compendium of 30 protein binding maps. Figure SM1 shows histograms of the identified BRICK sizes for various values of γ.

FDR can be defined in two ways:

- FDR_{BRICKs} = average number of BRICKs detected in 1,000 randomizations / average number of BRICKs detected in the 30 protein maps
- FDR_{probes} = average number of probes located in BRICKs detected in 1,000 randomizations / average number of genes in BRICKs detected in the 30 protein maps

The average number of BRICKs or probes represents the average number that we pick up per binding profile or per genome permutation. We settled on $\gamma = 1x10^{-4}$, which yields FDR_{BRICKs} = 2.5% and FDR_{genes} = 1.6%. Both FDR values are well below 5%, representing a stringent cutoff.

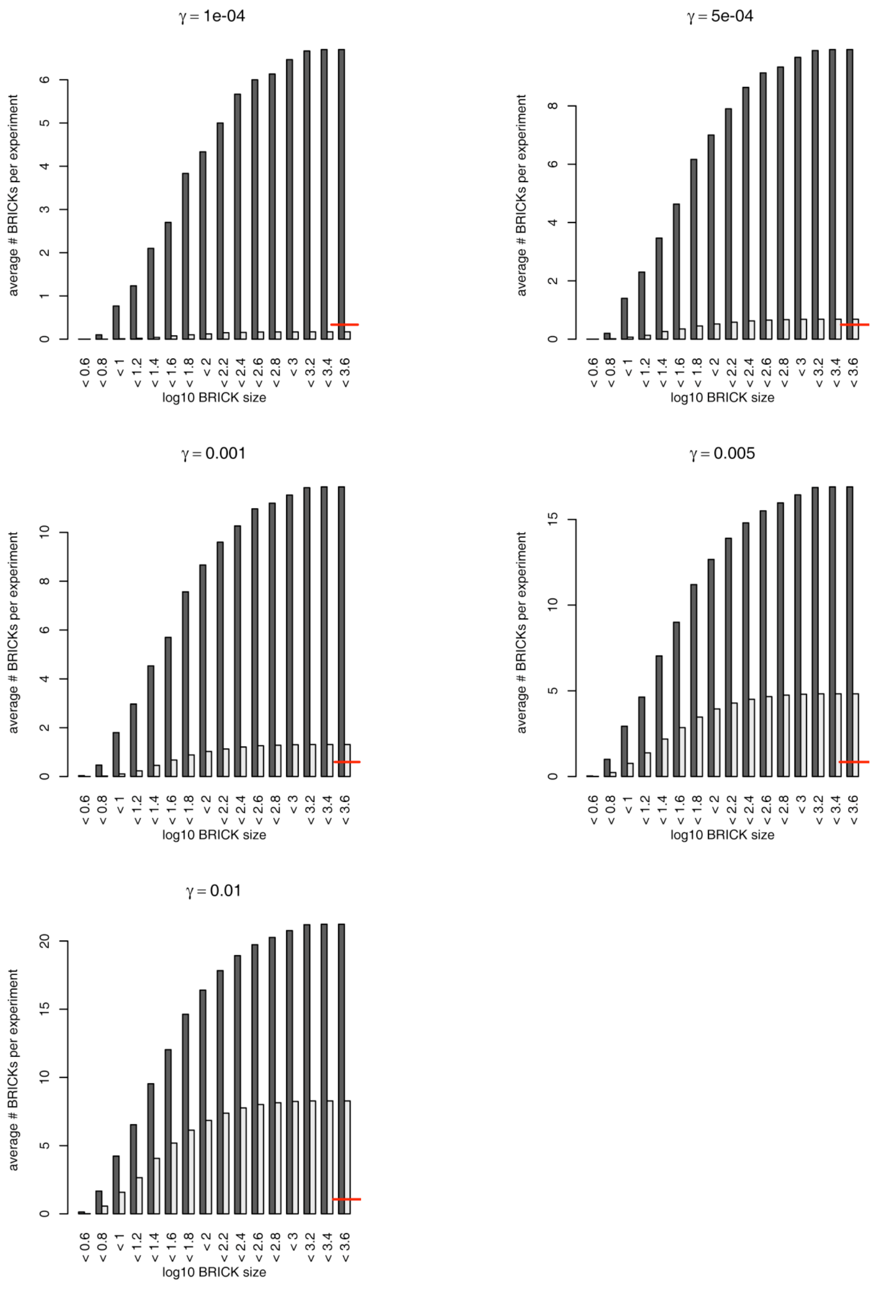

Figure SM1: Cumulative histograms of BRICK sizes for binding profiles and randomized data. A-E) Cumulative histograms show size distribution for BRICKs in the 30 binding profiles (black bars) and in 1000 randomized genomes (grey bars). γ values of 0.01, 0.005, 0.001, 0.0005 and 0.0001 were tested. Height of the bars indicates the average number of identified BRICKs of given size or smaller per experiment. The dashed red line denotes the FDR cut-off of 5%, for this given γ level. For γ=0.0001 the FDR is below 5%.

Performance of the BRICK identification algorithm on synthetic data

We further tested the reliability of the BRICK identification algorithm on a synthetic dataset that emulates a binding map of a hypothetical protein with a number of pre-defined chromosomal domains of various sizes and with various levels of binding. The datasets that we used for the Q_i-transformed real protein binding maps, contain ~8,000 probes. Therefore we began with a set of 8,000 uniformly distributed quantile scores. The top 25% of these quantile scores were devided into 3 categories: the top 1% (strong binding), top 1%-10% (medium binding) and top 10%-25% (weak binding). We then constructed a synthetic chromosome arm of 1,200 genes. On this chromosome arm we placed seven domains consisting of 5-100 neighboring genes that were assigned quantile scores selected from one of these categories, i.e., domains consisted of either "strong", "medium" or "weak" genes. Genes between these domains were randomly assigned a value from the remainder of the quantile scores. Thus, we created a model of a chromosome arm with several somewhat "noisy" domains in an otherwise unstructured "noisy" background.

We then tested whether the domains on the synthetic chromosome could be identified by the BRICKs algorithm. This was repeated in 100 independent simulation runs. Figure S3A and S3B show an example of, respectively, a domanogram and the actual quantile scores in one simulation. Figure S3C shows the BRICKs that were identified in each of the 100 simulation runs. Recovery of domains is generally reliable, although it depends somewhat on the domain size and signal levels in the domains. Domains of size 30 consisting of medium genes and domains of size 10 consisting of strong genes are always detected. Domains consisting of 100 weak genes or 5 strong genes are detected most of the times (~75-80% recovery). Domains consisting of 10 medium genes are rarely detected (12% recovery), and domains consisting of 20 or fewer weak genes are not recovered at all using our method. Importantly, all identified BRICKs corresponded to pre-defined domains, underscoring the low FDR of the algorithm. Only in rare instances (3 out of 100 simulations) are two neighboring domains detected as one large BRICK. Thus, the large BRICKs that were identified in many of the DamID and ChIP-on-chip maps are not the result of an intrinsic tendency of the algorithm to merge neighboring domains, but are likely to represent true biological domains. Taken together, these results indicate that our algorithm reliably detects domain organization in a noisy background.

Co-expression analysis

Developmental expression data was taken from ref. [1]. On this array, every exon in the genome is represented by a probe. Probe intensity values were log-transformed. For genes with multiple exon probes a mean intensity per gene was calculated. To avoid biases in correlation, the data for every developmental stage was variance normalized (mean of 0 and unit variance). Using this dataset we calculated the average pairwise Pearson correlation (APC) across all possible pairs of genes in a BRICK. For visualization purposes however, we need to correct the APC, since it decreases with the size of the window. For a window of n genes we scaled the APC using a scaling factor S_n. S_n was determined as follows. From the total set of genes we select n random genes. We calculate the APC for this subset of genes. We do this selection 1000 times for a given n. S_n is now the standard deviation over 1000 APCs.

Neighboring genes frequently show coregulation [1,2]. Because BRICKs obviously encompass neighboring genes, we want to determine whether, on a genomic scale, BRICKs are enriched for coregulated genes. We do this by comparing the expression patterns of the genes in BRICKs to the expression pattern of all other same-sized windows in the genome. However, for this analysis it is important to keep in mind that the BRICKs show hierarchical and overlapping organization (outlined in Fig. SM2A). To guarantee that the APCs are independent observations, we include a specific correlation between two genes only once in our analysis. This is achieved in the following manner. In figure SM2A, BRICKs 1 (yellow) and 2 (green) are the smallest BRICKs in the region, and the APC for those BRICKs are calculated by taking the mean of the correlations in (the lower triangle of) the square. The red BRICK (3), however, overlaps with the two smaller BRICKs, therefore for the calculation of the APC, the correlation in the dark gray squares are not taken into account. This leaves only the correlations in the light gray structure (subtracted structure). For the black BRICK we repeat the same procedure; all the correlations that fall within a smaller overlapping BRICK are left out of the calculation of the APC. Only the white subtracted structure is calculated.

For every BRICK we compare the APC in the window or subtracted structure to the APC in all the same-sized windows or subtracted structures in the genome. From this we can determine the rank of the APC of the BRICK in the genome. The coregulation rank of the

BRICK is transformed to a quantile score (q_{BRICK}) by dividing by the number of windows. Figure S5 shows cumulative distribution plots of q_{BRICK} for all binding profiles. Under the null hypothesis that chromatin domains are not enriched for co-regulated genes, one would expect the distribution of q_{BRICK} to resemble a uniform distribution between 0 and 1. Deviation from the uniform distribution (gray dashed diagonal in Figure S5), represents enrichment of coregulated genes in the domains of a given protein. We test for this using a Kolmogorov-Smirnov test for deviation from a uniform distribution.

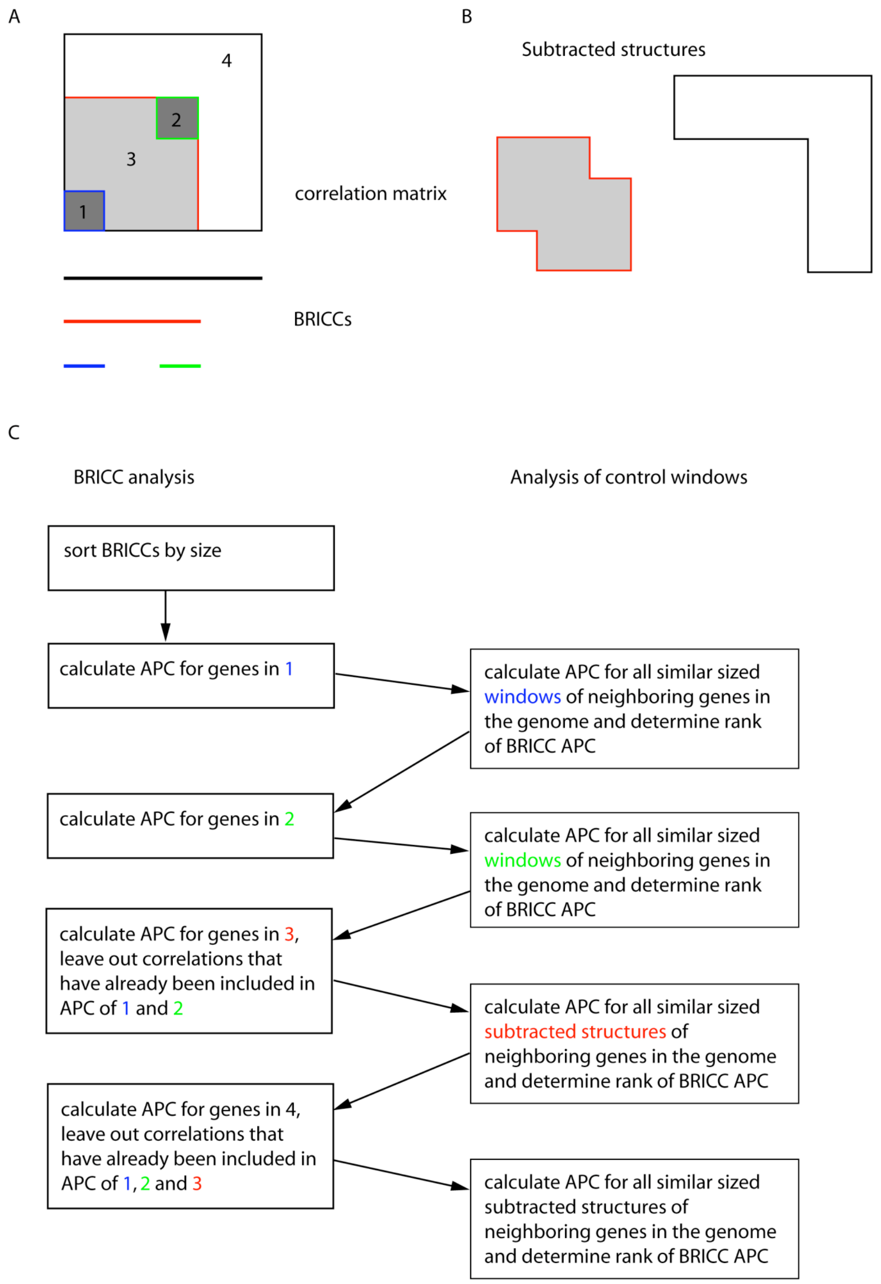

Figure SM2: Flow chart explaining the coexpression analysis. A) Partial correlation matrix depicting the correlation values that are included in the calculation of the average pairwise correlation (APC) for the shown BRICKs. B) For BRICKs that overlap with smaller BRICKs, correlations that have been used in the calculation of the APC of the smaller BRICK are not included, leading to the subtracted structures. C) Outline of the algorithm. For every BRICK a quantile score is calculated, representing the rank of the APC of the BRICK in a genomic context.

References

1. Stolc V, Gauhar Z, Mason C, Halasz G, van Batenburg MF, et al. (2004) A gene expression map for the euchromatic genome of Drosophila melanogaster. Science 306: 655-660.
2. Spellman PT, Rubin GM (2002) Evidence for large domains of similarly expressed genes in the Drosophila genome. J Biol 1: 5.
